# A High-Throughput Computational Framework for Identifying Significant Copy Number Aberrations from Array Comparative Genomic Hybridisation Data

**DOI:** 10.1155/2012/876976

**Published:** 2012-09-13

**Authors:** Ian Roberts, Stephanie A. Carter, Cinzia G. Scarpini, Konstantina Karagavriilidou, Jenny C. J. Barna, Mark Calleja, Nicholas Coleman

**Affiliations:** ^1^Department of Pathology, University of Cambridge, Tennis Court Road, Cambridge CB2 1QP, UK; ^2^Department of Biochemistry, University of Cambridge, Tennis Court Road, Cambridge CB2 1QW, UK; ^3^The Cavendish Laboratory, University of Cambridge, J. J. Thomson Avenue, Cambridge CB3 0HE, UK

## Abstract

Reliable identification of copy number aberrations (CNA) from comparative genomic hybridization data would be improved by the availability of a generalised method for processing large datasets. To this end, we developed swatCGH, a data analysis framework and region detection heuristic for computational grids. swatCGH analyses sequentially displaced (sliding) windows of neighbouring probes and applies adaptive thresholds of varying stringency to identify the 10% of each chromosome that contains the most frequently occurring CNAs. We used the method to analyse a published dataset, comparing data preprocessed using four different DNA segmentation algorithms, and two methods for prioritising the detected CNAs. The consolidated list of the most commonly detected aberrations confirmed the value of swatCGH as a simplified high-throughput method for identifying biologically significant CNA regions of interest.

## 1. Introduction

Correlating specific genomic copy number aberrations (CNA) with disease is an important and challenging first step in biomarker discovery [1]. Detecting CNAs that define genomic regions of interest using array comparative genomic hybridisation (aCGH) requires precise integration of probe signal amplitude, size (i.e., width) of copy number imbalanced region, and frequency of imbalance across a sample set, all referenced to relevant clinico-pathologic features.

There are two broad methods of aCGH data interpretation for biomarker discovery. The first, exemplified by the R Bioconductor package cghMCR [2], identifies regions showing the most frequent CNAs within a sample set, ranked by average signal amplitude. This approach to prioritization may under-call low prevalence high-level CNAs, such as homozygous deletions or gene amplifications that occur in small subsets of the samples analysed. The second method, targeted gene identification, exemplified by the genome topography scanning (GTS) algorithm [3] and Genomic Identification of Significant Targets in Cancer (GISTIC) module [4], is designed to localize regions of copy number imbalance most likely to be of functional significance. The GTS method models CNAs using parameters of signal intensity, region width and recurrence across a sample set, moderated by gene content. While this approach is able to identify significant regions of imbalance in heterogeneous samples, it relies on prior knowledge. GISTIC calculates the background rate of random chromosomal aberrations and identifies regions that are aberrant more often than would be expected by chance, with greater weight given to high amplitude events. Although gaining favour, a recent report notes GISTIC has trouble identifying relevant minimal regions of interest within larger tracts of CNA [5]. 

There are currently few open source methods for consolidating aCGH data across a set of samples. In addition, there are particular difficulties with handling large data sets derived from very high-density oligonucleotide-based aCGH platforms, where there may be a need to review many distinct significant regions of interest. To address these issues, we developed sliding windows adaptive thresholds CGH (swatCGH), a new computational framework for simplifying aCGH data analysis. swatCGH is a heuristic method based on strengths of the major existing approaches. It provides a robust systematic approach, which effectively automates the aCGH analysis process in order to identify CNA regions of interest and improve the reliability of candidate gene identification.

The framework is based on the analysis of average signal amplitude, region width and frequency of CNA occurrence, and enables these parameters to be identified as independent or associated events, including sample subset analysis by agglomerative hierarchical clustering. For each chromosome, swatCGH preferentially identifies regions that display the largest average signal intensity in the greatest proportion of the sample cohort.

The stages of swatCGH were designed to accommodate technical factors that may confound aCGH data analysis, particularly methods of signal intensity preprocessing, such as background correction, normalization, and classification of probe copy number states following segmentation [6, 7]. The R Bioconductor [8] based method enables application of multiple preprocessing configurations, probe segmentation algorithms, and classification strategies, in order to provide the most robust definition of significant CNA regions of interest. Uniquely, the approach also allows comparison and consolidation of analyses resulting from the various preprocessing methods used.

Here, we provide a detailed description of swatCGH. We exemplify the approach using a previously published aCGH dataset based on an analysis of 38 glioblastoma multiforme (GBM) samples using Agilent 44 K oligonucleotide arrays (GSE7602) [3]. The dataset had previously been analysed by GTS, leading to identification of functional redundancy between CDKN2A and CDKN2C tumour suppressor genes in GBM. We analysed the dataset by swatCGH, using data preprocessed with each of the four most frequently cited segmentation algorithms; circular binary segmentation from the package DNAcopy [9, 10], an adaptive weights smoothing method from the package GLAD [11], an homogeneous hidden Markov model (HomHMM) provided by the package aCGH [12], and a biologically tuned HMM (BioHMM) from the package snapCGH [13]. By consolidating data from the four analyses, we identified the most robust CNA regions of interest in the dataset. Based on our comparison of methods for prioritizing detected CNAs, we present results as a summarized list ranked by mean signal intensity, with web-style summary pages to facilitate data verification and efficient selection of candidate genes. In addition, the detailed report of all parameters analysed allows for thorough assessment of other potential regions of interest that are not recorded on the ranked list. By comparing our findings with the previous GTS study [3], we conclude that our heuristic framework offers a simplified high-throughput approach to defining novel genomic loci of potential clinical relevance.

## 2. Materials and Methods

### 2.1. Overview of Key Features of swatCGH Framework

swatCGH may be viewed as an aCGH informatics pipeline, in which the input comprises aCGH raw data files, experimental details, an array layout file, and a set of configurations describing the parameters to be used in the analysis. Because of the computational requirements of the methods, we used the high-throughput facilities of CamGrid, Cambridge University's federated computational grid, based on Condor middleware [14]. To permit automated distribution of the analysis, separate R jobs were generated, to perform discrete steps of preprocessing, segmentation, region definition, and reporting.

swatCGH has three important distinctive features. First, in order to reduce noise, we identified CNAs based on signal intensities of groups of neighbouring probes. We identified significant CNA regions of interest across a sample set, based on windows of fixed numbers of probes ranging from 3 to 20. For each window, we measured the percentage of samples within the set which showed the same aCGH copy number classification, a value referred to as the probe window score (PWS). We undertook sequential reanalysis following window displacement by one probe along the length of each chromosome, (i.e., using sliding windows). Data for all PWS across a sample set provides a measure of the overall prevalence of each CNA within the set. Second, we determined the most frequently occurring regions of interest across a sample set for each chromosome separately. Placing PWS in genome position order along a chromosome, we applied varying thresholds to the frequency of CNA occurrence across the sample set, in order to identify the most frequently occurring 10% of CNAs. Accordingly, chromosomes showing a relatively high frequency of CNAs required more stringent thresholds to identify the most commonly occurring 10% regions of interest. We refer to this process as applying adaptive thresholds (AT), an approach that enables identification of lower prevalence abnormalities that may nevertheless be highly significant in sample subgroups. Third, based on findings reported below, the identified CNA regions of interest were ranked by mean signal intensity (similar to cghMCR, [2]), ensuring that significant poorly annotated regions of the genome were not neglected.

Further details of these features of swatCGH are provided in the following sections. The published dataset used for exemplification (GSE7602) was chosen because it was derived from a relatively large number of well-characterised tumour samples, was based on a high-density oligonucleotide microarray platform, and had previously been analysed by GTS [3].

### 2.2. swatCGH Framework Applied to a Computational Grid

Supplementary Figure S1 presents an overview of our aCGH data processing framework, illustrating the integration of swatCGH into Condor CamGrid. Detailed descriptions of all R scripting methods are available online at doi:10.1155/2012/876976 (http://www.path.cam.ac.uk/research/investigators/coleman/swatCGH/). swatCGH is initiated on a local Condor submission node, with aCGH data being imported, compressed, and prepared for grid submission. Next, a single Condor job submits data to CamGrid for Batch mode R preprocessing using snapCGH. Essentially, this stage comprises data import, background subtraction, and normalization within arrays. Thereafter, a data interdependent Condor process is performed on a per segmentation method, per chromosome basis. This stage utilizes Condor's own directed acyclic graph manager (DAGMan) to schedule the linked jobs. The DAGMan stage is composed of three separate jobs, each defined by a single R script. First, array data is partitioned into separate autosomes, then imputation, segmentation, and classification of aCGH states is performed. Second, probe window scoring for a range of probe window sizes is undertaken. Third, swatCGH generates web-style reports of identified contiguous regions of interest. Following Condor DAGMan completion, a postprocessing stage finalizes the analysis by removing temporary files and consolidating the separate chromosomes into a fully linked report that describes CNA across the genome.

To ensure access to all required R Bioconductor libraries, we used a shared copy of R, served to CamGrid from a host running a chirp server. To enable the use of R batch mode on CamGrid the necessary process of generating discrete R scripts for each executed Condor job was undertaken via the use of template files. The process of turning a template file into a job specific script file was undertaken within the swatCGH shell wrapper, using *sed*, the Unix stream editor. While our implementation of the swatCGH framework utilized Condor CamGrid due to its local availability, we consider that modification of the framework for use with other distributed computational facilities and schedulers (e.g., Globus) would be a straightforward matter, due to the use of simple text configuration and template files.

### 2.3. Classification of Segmented Chromosomal Regions

We apply a 5-point classification scheme to array probes within segmented regions, comprising: high level loss; loss; normal copy; gain; high-level gain/amplification. In classification score tables, these states are represented by −2, −1, 0, 1, and 2, respectively. Classification is undertaken using the nudSegmentation algorithm (snapCGH and BayesCGH Bioconductor packages), which states that segments are copy number abnormal if their absolute computed fluorescence ratios are greater than the difference between the middle fifty of the distributions of normalised observed fluorescence ratios and the middle fifty of the predicted values, multiplied by an appropriate factor change (we used a default value of 75% factor change difference). nudSegmentation separates high-level CNA from single copy gain or loss based on region width, the upper limit of which was set at 10 probes (approximatly 700 Kb on the Agilent 44 K platform). 

### 2.4. Algorithm for Determining CNA Prevalence

**Figure 1:**
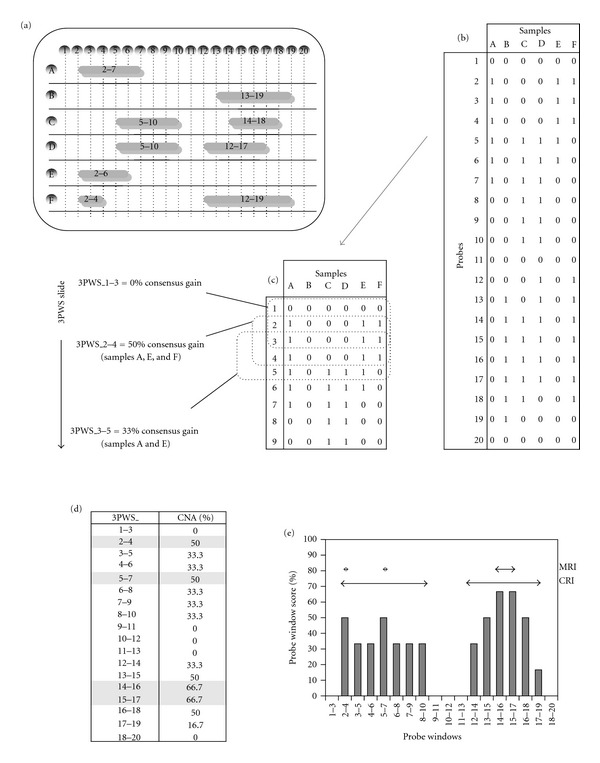
Illustration of the probe window scoring method. Panel (a) is a hypothetical aCGH experiment (samples A–F, probes 1–20). Horizontal bars are regions of copy number gain. Panel (b) is the same information in classification table format. Panel (c) shows scoring of the classification table for the first three of the 3-probe windows. Panels (d) and (e) summarise the scoring, with the graph plotting for each probe window (*x*  axis) the percentage of samples showing the same aCGH classification (i.e., probe window score). This identifies two CRIs (probes 2–10 and 12–19). The MRIs within each region are shaded in panel (d).

Figure 1 illustrates the processes involved in probe window scoring, using a hypothetical array experiment (Figure 1(a)) comprising 6 samples (A–F) hybridised to a 20 probe platform (probes numbered 1–20), where grey shaded horizontal bars indicate regions of gain. We define a continuous region of unbroken CNA, in which all probes are consensually imbalanced, as a contiguous region of interest (CRI). To identify CRIs, we first construct a classification score table (Figure 1(b)), in which probe gain is denoted by 1 and no change by 0. Had there been loss, deletion, or amplification, scores of −1, −2, or 2 would have been recorded. Figure 1(c) illustrates probe window scoring for the smallest window size of three probes. Each window receives a score that indicates the proportion of samples in which the same aCGH classification (gain or loss) is seen for all probes within the window. In this exercise, gain is combined with amplification and loss with deletion. For example, no sample shows gain of all probes in the first window (probes 1 to 3), hence 3PWS_1–3 = 0%. The rectangle slides one probe down, and samples A, D, and E all now share consensus gain for probes 2–4, hence 3PWS_2–4 = 50%. The process repeats until all probe windows have received a score (Figure 1(d)), after which a prevalence plot (Figure 1(e)) summarises the discrete regions of gain identified. The plot (Figure 1(e)) is intersected with an AT in order to select CRIs that occur above a given frequency within the sample set. In general, AT values are set for each chromosome to identify the 5% that contains the most commonly occurring copy number gains and the 5% that contains the most commonly occurring copy number losses. Within each CRI, we define the smallest region of probes showing the most frequent concordant CNA across the sample set. Such a region is referred to as a minimum region of interest (MRI). By definition, a CRI will contain at least one MRI, although it may contain more than one MRI. For CRIs that show no variation in frequency of CNA between probes, the MRI and CRI will be the same.

We compute PWS for window sizes ranging from 3 to 20 probes, corresponding to approximately 210 Kb ~ 1.4 Mb on the Agilent 44 K platform. Calculating CNA recurrence across probe windows effectively provides technical replication that smoothes point fluctuations introduced by technical error, non-specific binding, or copy number variations affecting discrete oligonucleotide probes. We consider that PWS from larger window sizes will more robustly reflect recurrent CNAs within a sample set, and be less susceptible to noise. swatCGH therefore requires that CNA regions of interest detected in larger window sizes are also present in internal smaller windows, at equal or higher frequencies of recurrence. This process is exemplified in Supplementary Figure S2, for the deletion mapping of the CDKN2A locus on chromosome 9, based on the published GBM dataset [3]. Here, analysis using a 20 probe size window shows a discrete region of loss on 9p. Reanalysis with 5-probe windows and 3-probe windows confirms the significance of the CNA and focuses the MRI to 21.73 Mb–22 Mb.

### 2.5. Chromosome-Specific Adaptive Thresholds Delimit Regions of Interest

Considering each chromosome separately, we apply decreasing ATs to delineate the 5% of the chromosome that contains most frequently occurring regions of copy number gain and the 5% that contains most frequent regions of copy number loss (10% overall CNA). This strategy normalizes the CNA detection process across chromosomes and for different segmentation algorithms, and allows lower chromosome specific prevalence CNA to be determined. Supplementary Figure S2D illustrates application of an 80% AT to gate CNA regions of interest on chromosome 9. We use the following rules for selecting the ATs, which are applied independently to regions of copy number gain and copy number loss on each chromosome. (1) We select the lowest threshold that results in approximately 5% CNA; (2) computing % CNA across the chromosome, ATs are selected in sequence from 80% to 20%, in decreasing 10% steps. If an AT results in 0% CNA, the next higher threshold is accepted, even if CNA > 5%; (3). Where the lowest stringency threshold (i.e., 20% frequency across the sample set) results in 0% CNA, the chromosome is deemed to be without CNA.

**Figure 2:**
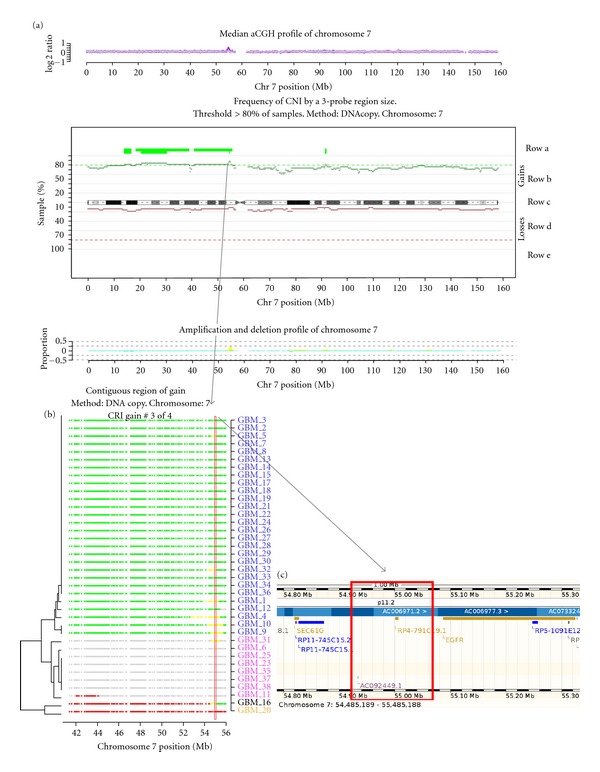
Identification of regions of interest in GBM dataset by swatCGH. The panels show representative swatCGH data for chromosome 7, showing copy number gain using data segmented by DNAcopy. Panel (a) shows the chromosome overview plot, representing the median aCGH profiles (top), sample recurrence plot (middle), and high-level CNA plot (bottom). Dashed lines in the middle panel represent 80% AT. Panel (b) is regional probe classification view of a copy number gain CRI on chromosome 7 (42–56 Mb). In panel (b) samples are clustered by probe classifications (green = gain; red = loss; grey = normal; yellow = high-level gain; cyan = high-level loss). The red box indicates the MRI identified for the region, linked by the arrow from the same region of the middle plot in panel (a). Panel (c) illustrates a hyperlink from the MRI to the ENSEMBL genome browser, provided in swatCGH output.

Figure 2 uses the GBM dataset to demonstrate the process of selecting CNA regions of gain on chromosome 7 (EGFR locus; Figures 2(a), 2(b), and 2(c)), while also illustrating aspects of swatCGH graphical output. An example of detecting CNA regions of loss is given in Supplementary Figure S3 (chromosome 9; CDKN2A). In Figure 2, panel (a) shows a chromosome overview plot, which summarises chromosome CNAs, and represents the starting point for identification of regions of interest. The overview plot is composed of three parts. The upper panel is the median aCGH profile of all arrays, where purple margins are 95% confidence intervals. The centre panel is a sample recurrence chart, which shows probe window score by chromosome position (using 3-probe windows), and indicates the frequency of CNAs across the sample set. The ideogram axis is at the centre (row c), with the frequency of CNAs for individual probe windows being shown by narrow bars above and below. Probe windows showing copy number gain are in green above the ideogram (in row b), while windows showing copy number loss are in red below the ideogram (in row d). Where adjacent 3-probe windows show the same frequency of CNA, they merge to form longer bars.

Aligning the median aCGH plot (upper panel in Figure 2(a)) with the frequency of recurrence across samples (middle panel in Figure 2(a)) provides a useful method for readily identifying CNAs most likely to be significant. In the analysis shown in Figure 2(a), an AT of 80% was required, hence the gated CRIs represent probe window spans that are gained or lost in ≥80% of the sample cohort. These gated CRIs are summarised as thick green bars for gain (row a) and thick red bars for loss (row e). All CRIs are numerically indexed, for subsequent cross referencing. For example, chromosome 7 copy number gain CRI#3 (containing the EGFR locus) is shown in detail in Figure 2(b). Finally, the lower panel in Figure 2(a) is a frequency plot of high-level CNAs (gains and losses), maximally scaled to 25% of the sample set. In swatCGH analysis, plots of CRIs by genome location, and sample-based views of all indentified MRIs are generated by default and presented as web-style reports. Supplementary Figure S4 provides a genome wide overview of CNAs in the 38 sample GBM data set following segmentation by DNAcopy.

### 2.6. Reviewing Significant Regions of Interest

A major difficulty in aCGH analysis is identifying CNAs that are most likely to target genes of functional importance. To assist rapid selection of such regions, swatCGH produces web-style summary pages for each chromosome, for each method of data segmentation, at a selected range of probe window sizes. Summary pages provide links to all processed data, supporting verification of the selected regions. The reports provided comprise chromosome overview, copy number karyograms, sample clustering by regions, regional probe classifications, and supporting data in tabular format. Unsupervised hierarchical clustering is undertaken using the classified aCGH call scores within the gated CRIs for each chromosome, in order to demonstrate any sample-dependent CNA patterns. The fact that the top 10% most frequently occurring CNAs are defined for all chromosomes ensures a detailed CNA profile for each sample and prevents significant low recurrence CNAs from being missed. This approach is likely to enrich for genes or genomic regions that mediate phenotype variation across clinico-pathological subgroups. Finally, swatCGH generates a table of CRI data ordered by genome position, with each row representing a discrete region. Rows are serially indexed, and maintain the indexed order of CRIs shown in the chromosome overview plots (Figure 2(a) and Supplementary Figure S3A). Following a region hyperlink reveals the aCGH classification of all probes in the region, as illustrated for one CRI in Figure 2(b). In addition, hyperlinks to on-line genome databases are also provided (Figure 2(c)).

## 3. Results

### 3.1. Comparison of CNA Detection following Four Segmentation Algorithms

All parameters relevant to swatCGH analysis of the GSE7602 dataset are provided in a single plain text file (Supplementary Text File 1). We demonstrated the performance of swatCGH by analysing the published GBM aCGH data, following the application of the segmentation algorithms DNAcopy, GLAD, HomHMM, and BioHMM (using developer-recommended default parameters), scoring window sizes of 3–20 probes. CRIs in individual chromosomes were identified based on adaptive thresholds of 20%–80% of the samples analysed, in order to identify the 10% most frequently occurring CNAs. 

Using unfiltered data for the 38 GBM samples, we observed that BioHMM and HomHMM led to detection of a greater number of discrete CRIs than DNAcopy or GLAD (Table 1, italic columns). However, while BioHMM and HomHMM led to identification of percentages of the genome showing CNA that were similar to the 10% target of adaptive thresholding (10.96% and 8.89%, resp.), the CRIs detected following DNAcopy and GLAD represented 25.17% and 31.08%, respectively. The latter methods identify relatively large CRIs and more stringent ATs led to <10% of the genome being detected as showing CNAs. Interestingly, when comparing the ratios of the sizes of the DNA regions identified as showing copy number gain to those showing copy number loss, BioHMM led to detection of a greater proportion of gain (ratio 2.03), while DNAcopy led to preferential identification of loss (0.64). GLAD and HomHMM led to detection of similar intermediate ratios of gain to loss (1.62 and 1.55, respectively).

### 3.2. Amplitude-Dependent Prioritization of Detected CNAs

We investigated two approaches to prioritization of the regions of interest derived from swatCGH analysis. First, we filtered MRIs using a modification of the signal amplitude dependent method of Aguirre et al. [2]:

(1) f(x)=e−(x−μ)2/(2σ2)σ2π.

Briefly, a probability density function (1) was computed using a permutation approach from mean signal intensities (*μ*) with scale parameter (*σ*) for each probe window size employed, with sampling size in the probability distribution being weighted for chromosome length. MRIs were filtered for regions demonstrating a statistically significant deviation in mean signal intensity (mean log 2 ratio across all arrays, *P* < 0.1). The number of CRIs that contained a significant MRI is shown in Table 1, roman columns. For data preprocessed by all DNA segmentation methods, we observed generally proportional reductions in the number of CRIs detected, compared to those identified from the unfiltered data. Interestingly, however, all segmentation methods now led to detection of similar percentages of the genome showing CNAs (1.22%–1.89%; Table 1 roman columns).

**Figure 3:**
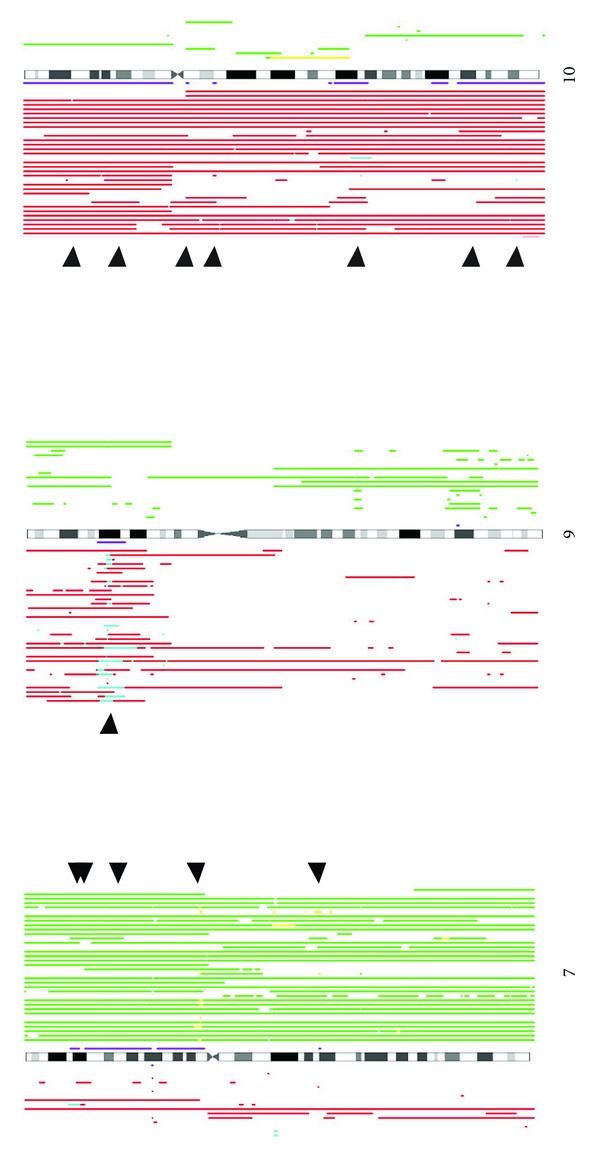
Representative swatCGH copy number karyograms. The results shown are for chromosomes 7, 9, and 10, using data segmented by DNAcopy. Red bars at the bottom of each ideogram indicate regions of copy number loss, while cyan bars signify high-level loss. Green bars at the top of each ideogram represent regions of copy number gain, while yellow bars represent high-level gain. The purple bars on either side of each ideogram denote the CRIs identified by swatCGH analysis. Arrowheads locate MRIs identified as significant using amplitude based prioritization (*P* < 0.1).

The regions of interest identified from 3-probe window data are shown in Supplementary Tables S1A (copy number gain) and S1B (copy number loss), ordered by significance value of MRIs. These data illustrate the value of AT setting in identifying lower prevalence CNAs. For example, using Supplementary Table S1A DNAcopy data as reference, the 80% AT value required to achieve ~5% CNA gain on chromosome 7 (SEC61G, 89%) would entirely eliminate gains determined on chromosome 4 (CHIC2) that had a maximal prevalence of 32% and required 20% AT to achieve 5% CNA. While all segmentation methods led to identification of chromosomes 7 and 9 as the regions of most frequent copy number gain and loss respectively, there were discrepancies in the regions lower in the ranked lists. For example, BioHMM and GLAD led to identification of gain on chromosome 1, while HomHMM led to detection of gain on chromosomes 8 and 17. Similarly, only BioHMM led to identification of loss on chromosome 4, while other methods led to detection of loss on chromosome 1. Intersection of the methods suggest chromosomes 3, 4, 5, 7, and 20 are the most common sites of copy number gain, while chromosomes 1, 9, 10, 11, 13, and 14 are the most common sites of copy number loss. DNAcopy led to identification of this CNA profile most closely (summarized in Supplementary Figure S5).  Figure 3 shows CNA on three of these target chromosomes using the copy number karyogram format of DNAcopy analysis. At high resolution, all segmentation methods led to mapping of the top copy number loss MRI to the CDKN2A locus (chromosome 9; 21.87 Mb). DNAcopy led to mapping the top copy number gain MRI to RP4-791C19, a clone located mid-way between SEC61G and EGFR. The remaining methods led to mapping the top region of gain precisely to SEC61G (chromosome 7; 54.9 Mb).

### 3.3. Gene-Centred Prioritization of Detected CNAs

The second method used to prioritize the MRIs derived from swatCGH was a modified version of GTS [3], which moderates average signal intensity by frequency of occurrence across a sample set and also incorporates weighting for gene density:

(2) ARI=log⁡⁡ 2(MRI  signal  intensity¯)×recurrence,  

(3) RIC=Number  of  genes  per  MRINumber  of  probes  per  MRI,

(4) AFI=(RIC×ARI)ARI,

(5) GDW=AFI×ARI.

For this approach, we generated values for average aberration recurrence index (ARI; mean aberration log 2 ratios multiplied by recurrence, (2)) and aberration focality index (AFI) as originally described [3]. To estimate regional information content for AFI calculation, we determined the regional information content (RIC), being the number of genes present within an MRI divided by the number of probes comprising the MRI (3). The product of ARI and AFI (4) gave a prioritization score, gene density weighting (GDW), which reflected not only average signal intensity but also recurrence and information content (5). This gene-centred prioritization method was applied first to the list of MRIs filtered by the amplitude-dependent prioritization method (i.e., those in Supplementary Table S1) and resulted in the reordering shown in Supplementary Table S2.

Using this modified GTS approach, BioHMM and GLAD again led to identification of SEC61G as the top gained locus, while HomHMM led to SEC61G being placed second, behind SKAP2. DNAcopy did not lead to identification of SEC61G gain, instead EGFLAM was identified as the top gained locus. In addition, the lower placed loci were reordered. The majority of the segmentation methods now led to elevation of EGFLAM (chr 5), EDN3 (chr 20), and CHIC2 (chr 4) above minor placed chromosome 7 loci, which dominated in the list ranked only by amplitude (Supplementary Table S1). Changes were also seen in the ranking of regions of copy number loss. Whereas regions on chromosomes 9 and 10 dominated, CDKN2C and FAF1 now ranked above CDKN2A after analysis following GLAD and DNAcopy segmentation.

We next applied the gene-centred prioritization method to the unfiltered MRI list, to test whether any MRIs had been excluded by the initial amplitude-based filtering step. We selected the top 10 MRIs identified from unfiltered data processed by each segmentation method, for comparison with data from the previous analysis (i.e., the data shown in Supplementary Table S2). Of the 80 MRIs so selected (Supplementary Table S3), 34 would have been excluded by amplitude-dependent filtering (i.e., *P* > 0.1). In addition to previously reported region listings, BioHMM now led to identification of copy number gain at loci on chromosomes 16 (CDH11; IRX5) and 17 (MAPT), and copy number losses at loci on chromosomes 1 (NPH4) and 15 (SPRED1). DNAcopy led to identification of gains on chromosomes 2 (Y_RNA), 16 (CDH11, CDH9, and IRF8) and 17 (MAPT), and losses on chromosomes 13 (SLITRK5; CYSLTR2), 14 (EGLN3; NPAS3), 15 (SPRED1), and 18 (TCF4; SNORA73). GLAD led to identification of gains on chromosomes 16 and 12, and losses on chromosomes 12, 13, 15, and 18, while HomHMM led to identification of additional gains on chromosomes 3 (TPRG1), 8 (EXT1), and 1 (FCRL5; NBPF15) and losses on chromosomes 12 (NAV3) and 11 (DSCAML1).

Based on these observations, we conclude that prioritization by amplitude favours the highest frequency CNAs that also display largest signal amplitude within the sample set, for example, gains on chromosome 7 and losses on chromosomes 9, and 10, regardless of whether the regions are gene-coding. In contrast, prioritization by recurrence-moderated average signal intensity, weighted for gene density, favours either gene dense regions, or large genes in smaller regions of imbalance, where the ratio of genes to probes will be disproportionately low. 

### 3.4. Integration of Ranked Regions of Interest

**Figure 4:**
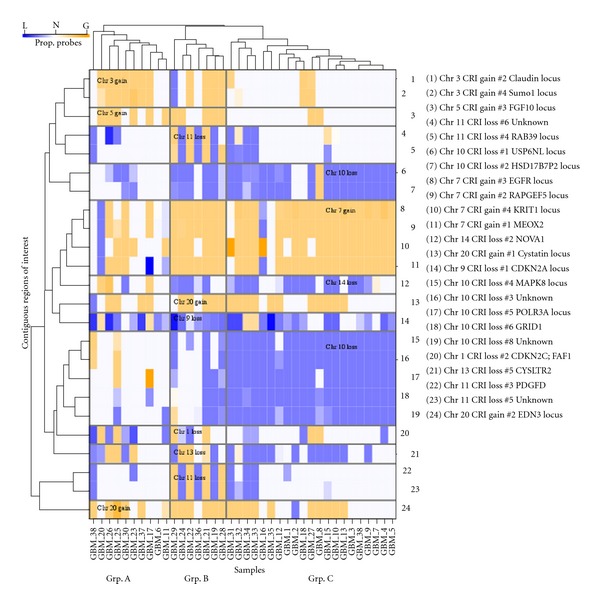
Hierarchical clustering based on selected CRIs. Each column represents a sample, while each row represents a CRI. The key provides details of each CRI, including the index number given during swatCGH processing. Shading denotes the proportion of probes in each CRI that demonstrated copy number gain (orange) or copy number loss (blue).

Finally, we combined all previous analyses to identify the most consistently detected CNAs in the dataset. For each of the two prioritization methods, we consolidated regions detected by the four segmentation algorithms into a consensus list of MRIs, requiring at least two segmentation methods to independently flag a region (Supplementary Figure S6). The gene-centred prioritization analysis used for this exercise was performed using data previously filtered by the amplitude-based prioritization method (Supplementary Table S2). We identified regions present in both lists to produce an overall set of 35 MRIs that we consider most robustly describes CNAs in the dataset (Supplementary Table 4). We performed agglomerative hierarchical clustering based on probes in the 35 CRIs that encompassed the 35 MRIs, using data that had been segmented with DNAcopy. CRIs were required to be detected above an AT of 40%, hence the CHIC2 locus was excluded. The sample set clustered into three subgroups (Figure 4). Notable characteristics included chromosome 3 gains in Group A, gross CNA on chromosome 11 in Group B, and losses on chromosome 10 in Group C. At locus level resolution (based on the MRIs), we observed CDKN2A loss across all groups, GRID1 loss predominantly in Group C, and elevated frequency of CDKN2C loss in group A (See Figure 4 key). The region of chromosome 7 spanning MEOX2 (15.7 Mb) to EGFR (55.1 Mb) was generally gained in all groups. There was increased frequency of Claudin and SUMO1 gain in Group A, FGF10 gain in groups A and B, and striking heterogeneity of PDGFD copy number in group B.

### 3.5. Initial Comparison with GISTIC

In a parallel analysis, we determined the proportion of genome deemed to show CNA by GISTIC for each of the four segmentation methods. The findings are provided in Supplementary Table S5 and may be compared directly with those in Table 1. Whereas the italic columns of Table 1 report the number of CRIs and the proportion of genome in CNA by swatCGH, Supplementary Table S5 reports the number of genes and proportion of genome called in CNA as determined from “wide peak intervals”. For swatCGH the average total proportion of genome in CNA was 1.5% (range 1.2–1.9%). The equivalent value by GISTIC was 2.8% (0.35–3.9%), with HomHMM generating an outlier value of 0.35%. The broader regions of CNA identified by GISTIC resulted in a list of ~400 candidate genes. In contrast, for swatCGH the prioritized CRI list was between 18 and 113 significant regions, resulting in a list of ~200 candidate genes across all segmentation methods (Supplementary Table S4). 

## 4. Discussion

swatCGH was designed as a simplified approach to selecting CNA regions of interest from aCGH data. This open source method enables consolidation of data across a sample set and can accommodate the large information content of high-resolution analyses, where theoretical limits extend beyond millions of probes by thousands of samples as defined by R data frame properties (see R documentation at http://cran.r-project.org/). The method incorporates sliding windows, as signal intensities estimated from groups of neighbouring probes are less likely to be subject to noise perturbation than discrete probes. Adaptive thresholds applied on a per chromosome basis increase the probability of identifying lower prevalence abnormalities that may contribute to significant patterns of disease heterogeneity, paralleling an aim of GTS, but in contrast to methods such as GISTIC, that are weighted towards oncogene detection. Selection of prioritized candidate targets is not computed by integration across probe window sizes. Instead, users are able to select a results panel based on a window size most appropriate for the array probe density used, and review outcomes for a range of probe window sizes by navigation through the web-based CNA reports. In our approach, the process for ranking CNA regions of interest is driven by mean signal intensity, preventing omission of significant nonannotated regions of the genome, and supporting inclusion of important lower prevalence abnormalities. The overall method is robust, systematic, and customizable, with all parameters specified in a single text file. The reporting of all analysis steps undertaken enables ready evaluation of all genomic loci, not just those in the ranked lists.

In applying swatCGH to a GBM test dataset, we observed considerable differences in the CRIs detected after data was preprocessed with four different segmentation algorithms most likely reflecting differences in arithmetic approaches to segmentation used by each algorithm. Interestingly, BioHMM, HomHMM, and GLAD led to preferential detection of copy number gain compared to copy number loss. This may be related to the empirically observed technical bias that can occur following aCGH normalisation, which produces a reduction in the dynamic range of global signal intensity in regions of copy number loss, compared to regions of copy number gain [15]. Interestingly, DNAcopy led to identification of a greater amount of loss than gain, associated with detection of more loss and less gain than the other segmentation methods, consistent with observations in the original description of the method [9], and most likely reflects the approach used for change point detection.

The most significant MRIs identified following processing by all four segmentation algorithms were generally similar (Supplementary Tables S1–S4). Indeed, DNAcopy, BioHMM, and HomHMM all led to the identification of a large majority of our final list of 35 consensus MRIs (Supplementary Table S4). The two methods used to prioritize MRIs had effects on the resulting ranked gene lists. The published analysis of the dataset used GTS, augmented by prioritization of CNAs based on a combination of focality, amplitude, and recurrence across the sample set [3]. This led to the biologically important novel observation that CDKN2C is a frequently deleted tumour suppressor gene in GBM. When we analysed the data set using a prioritization method based on signal amplitude alone, CDKN2C loss was also highly ranked following data segmentation with DNAcopy (ranked 4th) and GLAD (ranked 2nd), although not following segmentation with BioHMM or HomHMM. Reordering the amplitude-prioritized data by GTS-based gene density weighting led to elevation of CDKN2C in ranked lists of data segmented by DNAcopy (new rank 2nd) and GLAD (new rank 1st), indicating that gene density weighting has the potential to add value in some settings. However, we also observed limitations of the GTS-based approach when considering copy number gain in the dataset. Using the signal amplitude method of prioritizing regions of interest, the top region of gain identified after BioHMM, HomHMM, and GLAD was SEC61G, while the top region identified after DNAcopy was RP4-791C19, which maps midway between SEC61G and EGFR. However, when the GTS-based method was applied to the ranked genes, the RP4-791C19 locus was not identified in the DNAcopy segmented dataset, as it is nongene coding. This observation illustrates how methods of interpreting CGH data that weight importance by genomic content are critically dependent on accurate probe mapping and annotation.

The agglomerative hierarchical clustering function of swatCGH detects significant relationships between regions of interest across samples. In the data set analysed, we identified a cluster (Group A) in which there was enrichment for CDKN2C deletion in association with the more widespread deletion of CDKN2A, mirroring the original published observation [3]. The relevant cluster group was also defined by gain of chromosome 3 loci in the absence of CNAs on chromosomes 10 and 11, features that may contribute further to the phenotype of tumours in the cluster.

In conclusion, swatCGH is a distributed high-throughput aCGH data analysis heuristic that facilitates identification of CNA regions of interest suitable for further genetic and functional investigations.

## Supplementary Material

Supplementary Text File 1. Parameters supplied to swatCGH for the analysis of the GSE7602 data.

## Figures and Tables

**Table 1:** Comparison of regions of interest identified by swatCGH following four methods of DNA segmentation. The values shown are derived from 3-probe window analysis of the GBM dataset, using adaptive thresholding to limit CNAs to 10% of the genome. For each segmentation method data is provided for regions of copy number gain, regions of copy number loss, and for the total CNA. *Italic columns * represent findings for CRIs using unfiltered data, while roman columns represent data for MRIs filtered for significance using amplitude-based prioritization (*P* < 0.1).

	All regions		Filtered regions (*P* < 0.1)	
	Number CRIs	Proportion CNA	Number CRIs	Proportion CNA

	BioHMM
Gain	*253*	**7.34%**	69	**1.33%**
Loss	*227*	**3.62%**	44	**0.56**%
Total	*480*	**10.96%**	113	**1.89%**
Gain : Loss		**2.03**		**2.40**

	GLAD

Gain	*66*	**18.91%**	11	**1.04%**
Loss	*65*	**12.17%**	7	**0.18**%
Total	**131**	**31.08%**	18	**1.22%**
Gain : Loss		**1.55**		**5.67**

	DNAcopy

Gain	*99*	**9.81%**	13	**0.81%**
Loss	*118*	**15.36%**	19	**0.99%**
Total	*217*	**25.17%**	32	**1.80%**
Gain : Loss		**0.64**		**0.82**

	HomHMM

Gain	*275*	**5.48%**	46	**0.93%**
Loss	*216*	**3.39%**	24	**0.29%**
Total	*491*	**8.87%**	70	**1.23%**
Gain : Loss		**1.62**		**3.20**
