# Supplementary material for: A High-Throughput Computational Framework for Identifying Significant Copy Number Aberrations from Array Comparative Genomic Hybridisation Data
Source: Adv Bioinformatics. 2012 Sep 13;2012:876976. doi: 10.1155/2012/876976 (PMC3449101; doi:10.1155/2012/876976)
Supplement: Supplementary file 1 — Supplementary Text File 1. Parameters supplied to swatCGH for the analysis of the GSE7602 data. [file 876976.f1.pdf]

Additional files

Supplementary Text File 1. Parameters supplied to swatCGH for the analysis of the GSE7602 data.

```
#################################
## Section 1                  ##
## Core File locations        ##
#################################
##
# 1a# swatCGH Home Directory
#/path/to/home

# 1b# R_scripts1:  Read in Raw data and generate MA object
01_prepareMAdata.R

# 1c# R_scripts2:  MA --> MA2
02_prepareMA2data.R

# 1d1# R_scripts3:  MA2/callsData.csv --> prunedCloneScores
02_ComputeFreqs.R

# 1d2# R_scripts4:  prunedCloneScores --> chr / region figures and tables
03_GenerateOutput.R

# 1d3# R_scripts5: internalFunctions for swatCGH
01_internals_v5.R

# 1e# Shell scripts1
Shell_scripts1.sh

# 1f# Shell scripts2
Shell_scripts2.sh

# 1g# Shell scripts3
Shell_scripts3.sh


################################
## Section 2                  ##
## Data Directories           ##
################################
##
# 2a# 1_GPR_files
1_GPR_files

# 2b# 2_Required_files
2_Required_files

# 2b1# for_1_swatCGH
for_1_swatCGH

# 2b2# for_2_SNAP
for_2_SNAP

# 2b2a# Targets file
targets.txt
```

2b2b# Clone information file
014693_D_20070207.gal

2b2c# Has SpotTypes file?
TRUE

2b2d#
SpotTypes.txt

2b3# for_3_MOLE
for_3_MOLE

2b3a# Default condor submission description file
01default_submit

2b3b# Default parrot_R_executable file.
02parrot_exe.sh

2b3c# Chirp server mountpoints file
03mountfile

2b3d# Node executable file
04node_exe.sh

2b3e# Condor server path to parrot file. Requires full path
/prg/biosoft/linux-bin/parrot-64

2b3f# Chirp server address

2b3g# Chirp served R mount point

2b3h# DAG description file
05dagID

2c# 3_Output_files
3_Output_files

2c1# 1_processed
1_processed

2c2# 2_summaries
2_summaries

3# Output graphics format
pdf

##
Flags for snapCGH
#######################################

4a# snapCGH source for read.ma images
genepix

4a1# snapCGH coumns list for read.maimages

4a1a# R
F635 Median

4a1b# G
F532 Median

4a1c# Rb
B635 Median

4a1d# Gb
B532 Median

4b# Specify reference name
ref

4c# Method for background correction
subtract

4d# Method for normalize
median

4e1# method for processCGH
NULL

4e2# grouping for processCGH
ID

4e3# min chromosome number for processCGH
1

4e4# max chromosome number for processCGH
22

4f# Segmethods as b,d,h,g,p
b,d,g,h

4g# file name prefix for MA objects
MAprocessed

4h# nudSegmentation factor change value
0.75

```
####################################
## Section 2                    ##
## swatCGH aCGH analysis settings ##
####################################
##
## 5.  Population frequency cut off ranges
# 5a#  min cut, as used by prunedScores function
```

0.1

5b# maxInterval -> used by cloneScores. Upper limit of wordSize
20

5c# Word size vector (integers separated by commas)
3, 5, 10, 20

5d1# Min cut threshold
0.2

5d2# Max cut threshold
0.8

5d3# Step for threshold sequence
0.1

5e# Number of cluster groups
4

5f# Cluster group density
6

Supplementary Figure S1. Overview of swatCGH data processing framework.

Supplementary Figure S2. Focusing regions of interest using decreasing probe window sizes.

The AT used (dashed line) defines the CRI shown by the red bars, with the MRI being denoted by the thickest part of the bar. Use of (A) 20, (B) 5, and (C) 3 probe window sizes focuses the copy number loss region of interest on chromosome 9 in the GBM dataset to 21.73 Mb – 22 Mb. Panel D illustrates the setting of an 80% adaptive threshold (x axis) for gating the amount of copy number loss (y axis) to 5% of chromosome 9.

Supplementary Figure S3. Identification of regions of interest in GBM dataset by swatCGH.

The panels show representative swatCGH data for chromosomes showing copy number loss (A-C) for the GBM dataset, using data segmented by DNAcopy. Panel A shows the chromosome overview plot, representing the median aCGH profiles (top), sample recurrence plot (middle), and high-level CNA plot (bottom). Dashed lines in the middle panel represent 0% AT. Panel B is regional probe classification views of a copy number loss CRI on chromosome 9 (21.7-22.7 Mb). In panel B samples are clustered by probe classifications (green = gain; red = loss; grey = normal; yellow = high-level gain; cyan = high-level loss).

The red boxes indicate the MRI identified for the region, linked by the arrow from the same region of the middle plot in panel A. Panel C illustrates a hyperlink from the MRI to the ENSEMBL genome browser, provided in swatCGH output.

Supplementary Figure S4. Genome wide overview of CNAs detected in 38 GBMs by swatCGH

The panels show swatCGH CNA data for automsomes of the GBM dataset using data segmented by DNAcopy. Within each panel, the top view shows the median aCGH profiles, the middle view gives CNA recurrence plots where thin rectangles are CRIs and thick rectangles are MRIs (green; gain, red; loss). The high-level CNA plot is represented at the bottom (yellow; amplification, cyan; deletion). Dashed lines in the middle panel represent ATs separately computed to achieve per chromosomal gains and losses at 5%.

Supplementary Figure S5. swatCGH copy number karyograms

The results shown are for chromosomes 1, 3, 4, 5, 7, 9, 10, 11, 13, 14, & 20, using data segmented by DNAcopy. Red bars to left of each ideogram indicate regions of copy number loss, while cyan bars signify high-level loss. Green bars to right of each ideogram represent regions of copy number gain, while yellow bars represent high-level gain. The purple bars on

either side of each ideogram denote the CRIs identified by swatCGH analysis. Arrowheads locate MRIs identified as significant using amplitude-based prioritization ($p < 0.1$).

Supplementary Figure S6. Selection of MRIs identified in data preprocessed by at least two segmentation methods.

The panels show representative data for chromosomes 1, 3, 4, 5, 7, 9, 10, 11, 13, 14, and 20 with (A) showing amplitude-dependent prioritized data (as in Supplementary Table S1) and (B) showing data from gene-centred prioritization of the amplitude filtered data (as in Supplementary Table S2). In each graph the x axis represents genome position and the Y axis is the MRI index number (ordered by genome position). The horizontal bars represent MRIs identified following each segmentation method, colour coded (see key for colour code). Regions of overlap between different coloured points represent MRIs identified by multiple segmentation methods. These are highlighted in Panel A, where purple stars show regions identified from the amplitude prioritization data only, while cyan stars show regions identified from data prioritized using both methods.

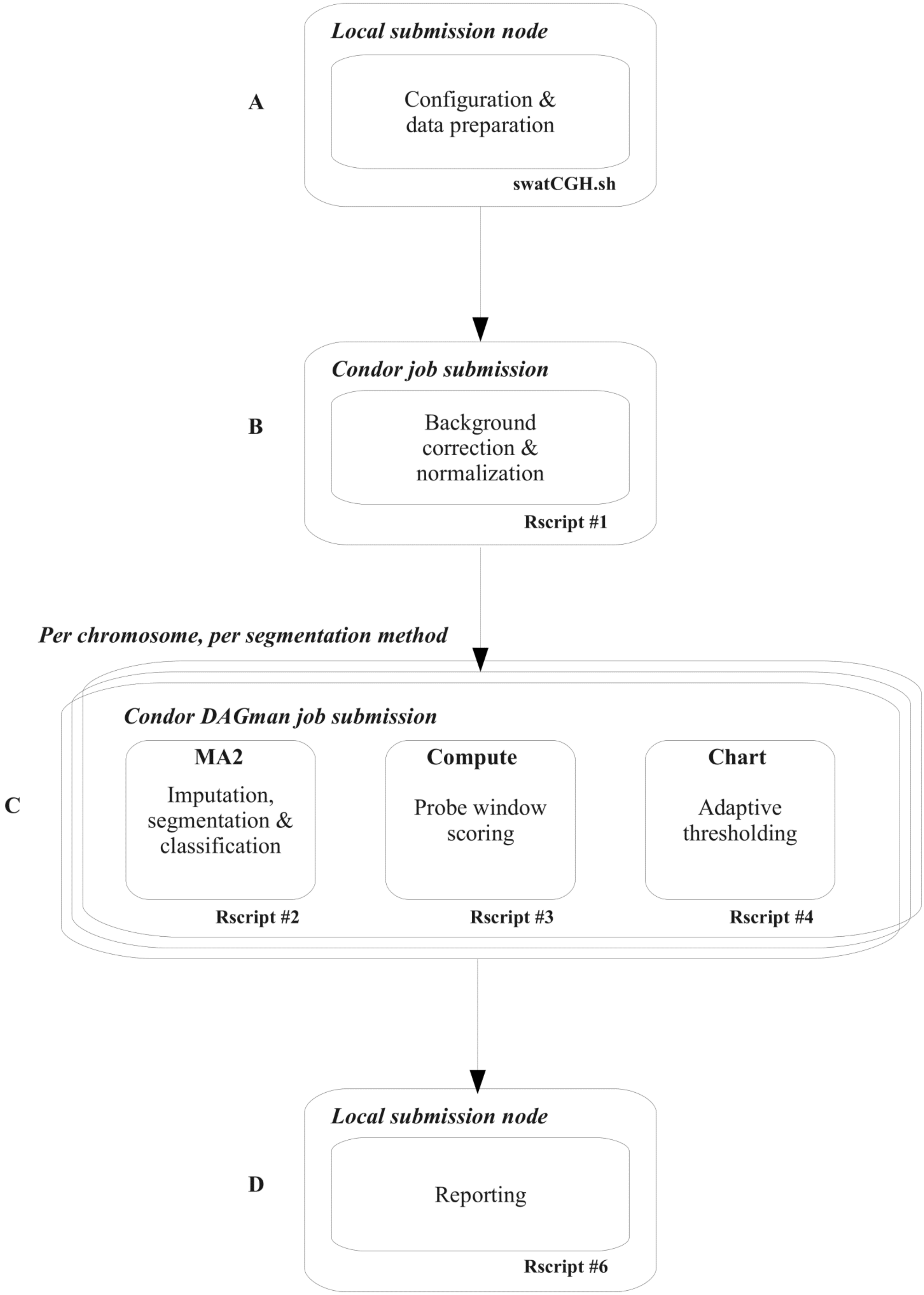

Supplementary Figure S1

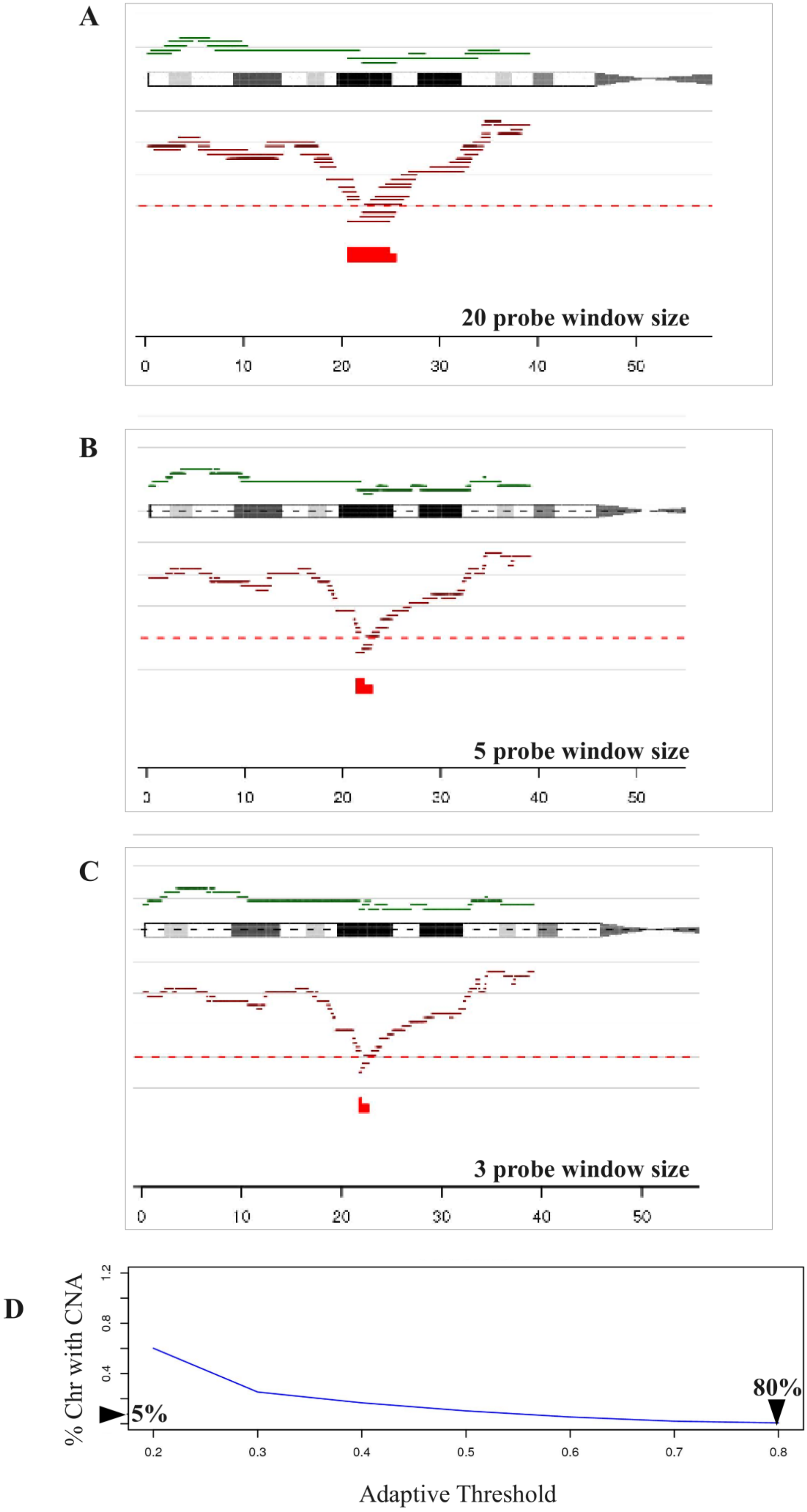

Supplementary Figure S2

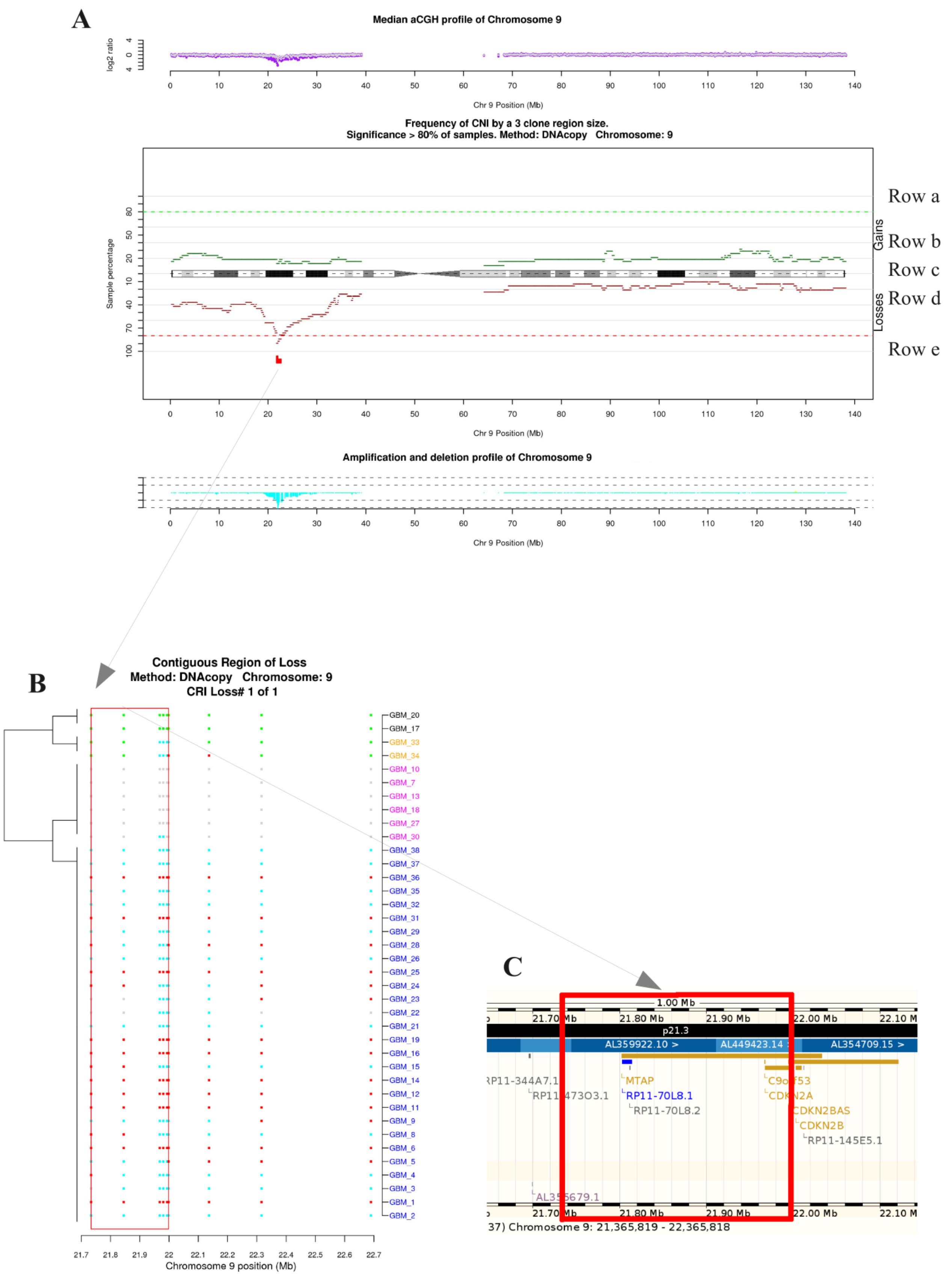

Supplementary Figure S3

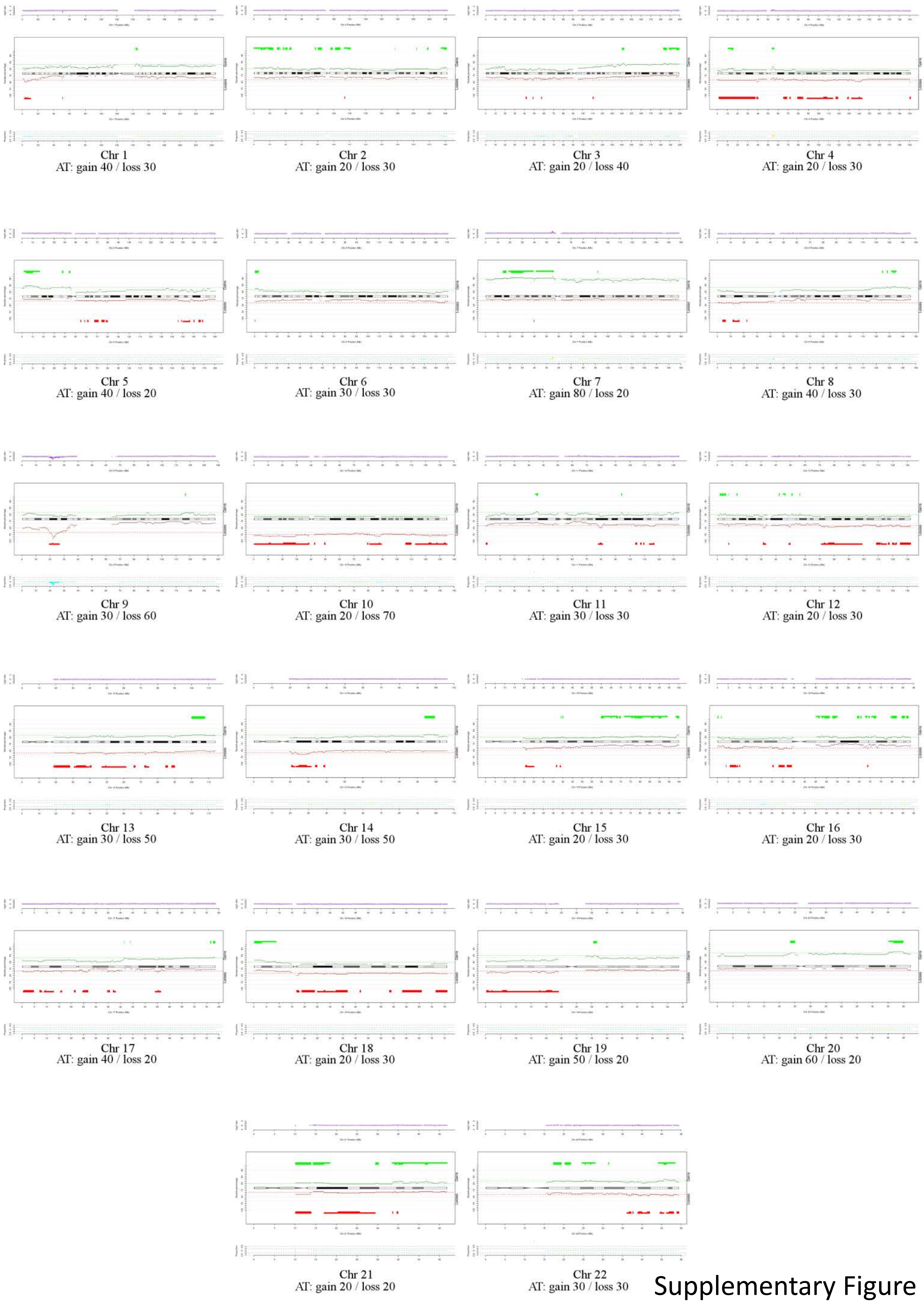

Supplementary Figure S4

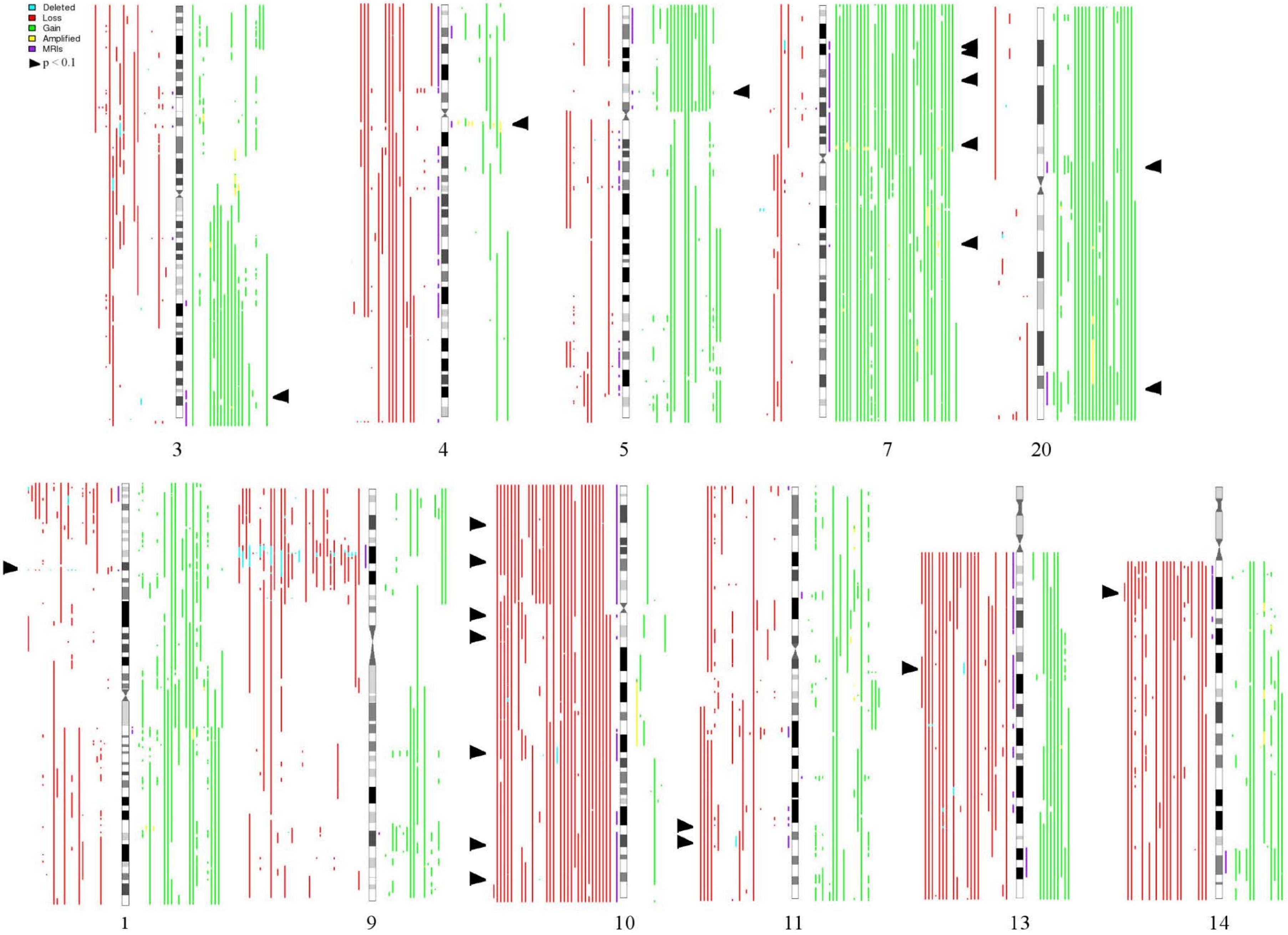

Supplementary Figure S5

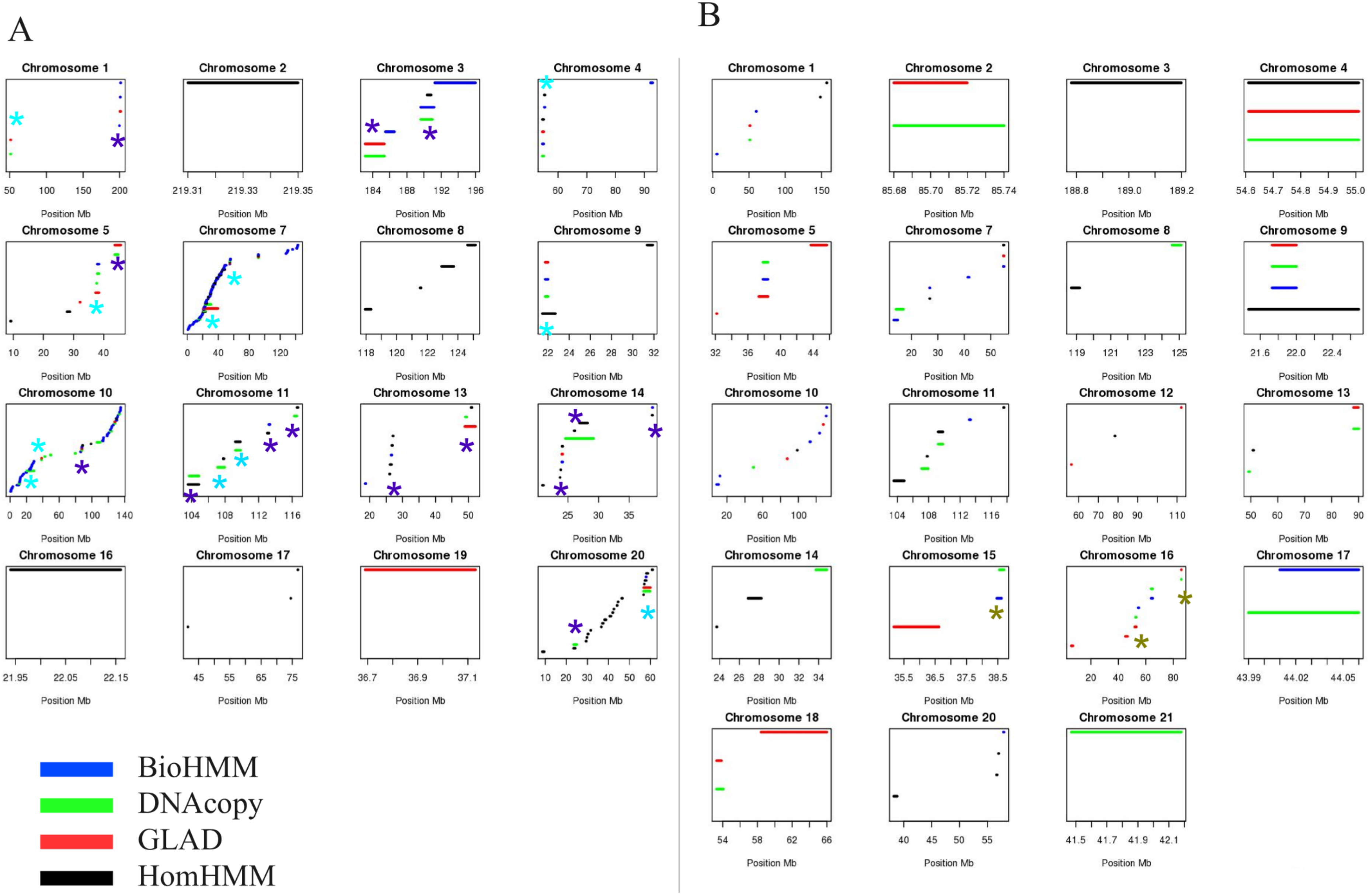

Supplementary Figure S6

Supplementary Table S1. Regions of interest detected by amplitude dependent prioritization following preprocessing using four methods of DNA segmentation.

Supplementary Table S1A shows data for copy number gain and Supplementary Table S1B shows data for copy number loss. Each section has four panels, representing data obtained following each of the four segmentation methods. Each panel is in halves. The left hand sections gives overall chromosomal statistics, including the CRIs identified, and the AT values used to detect them. The right hand panel reports the MRIs identified as significant following amplitude based prioritization, with respective gene information where appropriate. A maximum of 10 significant MRIs is given for any one chromosome. Significant regions are ranked by p-value following amplitude-based prioritization (p< 0.1). Column labels: Chr = chromosome; AT 5% = adaptive threshold to achieve 5% copy number imbalance; Number CRI = number of contiguous regions of interest; Mb DNA = combined length of CRIs imbalanced per chromosome; Prop. Chr. = proportion of chromosome forming CRIs; Max. PWS = maximum probe window score (i.e. frequency of CNA recurrence across the sample set); Recurrence = maximum recurrence frequency of an individual MRI across a sample set; Mid Position = location of MRI mid point; Mb DNA = width of MRI; GDW = gene density weight; Score = Modified GTS score; p-value = significance in amplitude based prioritization analysis; Gene = known genes in MRI. A maximum of 10 significant MRIs are given for any one chromosome.

Supplementary Table S2. Effect on rank ordering of significant MRIs identified by amplitude-dependent prioritization ($p < 0.1$), following application of gene-centred prioritization using modified GTS.

Findings are presented separately for data preprocessed by each segmentation method.

Supplementary Table S3. Effect on rank ordering of all MRIs following application of gene centred prioritization using modified GTS.

The table shows the top 10 MRIs identified from data processed using each segmentation algorithm.

Supplementary Table S4. The final set of 35 MRIs identified from the consolidated data.

The MRIs were identified following preprocessing with at least two DNA segmentation algorithms, for both amplitude-based and gene-centred prioritization methods. Column labels: Rated by = type of prioritization method, where (A) is amplitude-dependent and (B) is gene-centred modified GTS; Method = segmentation method, where (B) is BioHMM, (D) is DNAcopy, (H) is HomHMM, and (G) GLAD; CNA = type of copy number aberration, either gain or loss; Chr = chromosome; Start = location of region start (Mb); End = location of region end (Mb); Gene = known genes in region; * = Gene NA is approximately 80 kb from EGFR.

Supplementary Table S5. Comparison of regions of interest identified by GISTIC following four methods of DNA segmentation.

For each segmentation method, data is provided for regions of copy number gain, regions of copy number loss, and for the total CNAs.

Supplementary Table S1A (Copy number gain data)

Biologically Tuned HMM Gain

Chromosome Overall (All regions)						Minimal Regions of Interest (p < 0.1)						
**Chr**	**AT 5%**	**Number CRIs**	**Mb DNA**	**Prop. Chr**	**Max. PWS**	**Recurrence**	**Mid Position (Mb)**	**Mb DNA**	**GDW**	**Score**	**pValue**	**Genes**
7	80	40	46.04	28.93	0.89	0.894736842	54.897592	0.302918	5	6.1357	0.0000	SEC61G
						0.842105263	91.93295975	0.3053545	1.6	0.8729	0.0002	GATAD1; KRIT1; CYP51A1; hsa-mir-1285-1; Y_RNA
						0.868421053	26.9828035	0.056557	10	4.4991	0.0012	SKAP2
						0.842105263	30.6531605	0.168505	1.5	0.6894	0.0014	GARS; CRHR2
						0.842105263	30.97274125	0.3607235	1.667	0.7581	0.0015	FAM188B; INMT; GHRHR
						0.842105263	30.1675495	0.391869	1.5	0.6773	0.0016	ZNRF2; SCRN1; FKBP14; PLEKHA8; C7orf41; hsa-mir-550-1
						0.842105263	47.139409	0.17921	0	0	0.0016	NA
						0.842105263	29.851105	0.047752	3	1.3493	0.0017	WIPF3
						0.842105263	33.0358835	0.686869	1.333	0.5954	0.0018	KBTBD2; RP9P; FKBP9; NT5C3; RP9; BBS9; 5S_rRNA; SRP_euk_arch; hsa-mir-550-2
						0.842105263	48.1911105	0.148341	3	1.3378	0.0018	UPP1
						0.842105263	32.3211055	0.101359	3	1.3316	0.0019	PDE1C
4	20	13	6.74	3.53	0.34	0.342105263	54.806086	0.398224	2	1.7573	0.0104	GSX2; CHIC2; RPL21P44; snoU13
						0.342105263	55.3488785	0.197025	0	0	0.0182	NA
20	70	1	0.14	0.21	0.71	0.710526316	57.9437595	0.135107	7	2.635	0.0201	EDN3
1	30	29	15.03	6.03	0.39	0.368421053	200.937621	0.147022	1.25	0.6846	0.0628	CACNA1S; KIF21B; C1orf106; U6
5	50	1	0.61	0.34	0.5	0.5	38.163874	0.614326	8	3.1855	0.0654	EGFLAM
1	30	29	15.03	6.03	0.39	0.368421053	201.4081365	0.195149	0.667	0.3561	0.0678	RPS10P7; CSRP1; TNNI1; PHLDA3; LAD1; TNNT2
3	40	16	22.81	11.52	0.47	0.447368421	190.3889783	1.5647535	1.091	0.4303	0.0914	CCDC50; UTS2D; OSTN; IL1RAP; TMEM207; CLDN16; CLDN1; LEPREL1; TP63; 7SK; U6
						0.421052632	193.63141	4.740988	1.447	0.5957	0.0956	SDHALP1; VEZF1L1; TCTEX1D2; PCYT1A; Osta; ZDHHC19; TFRC; TNK2; MUC4; SDHALP2; PPP1R2; FAM43A; C3orf21; LSG1; TMEM44; GP5; HES1; CPN2; LRRC15; OPA1; HRASLS; C3orf59; FGF12; MUC20; Y_RNA; Y_RNA; U2; SRP_euk_arch; Y_RNA; SRP_euk_arch; U6; SRP_euk_arch; U1; snoU13; Y_RNA; hsa-mir-570; Y_RNA; 7SK
1	30	29	15.03	6.03	0.39	0.315789474	199.627756	0.036042	0	0	0.0993	NA
2	30	4	3.6	1.48	0.32							
6	30	4	2.39	1.39	0.32							
8	40	3	4.14	2.83	0.42							
9	30	3	1.42	1.01	0.32							
10	0	0	0	0	0							
11	30	5	4.98	3.69	0.37							
12	20	19	10.6	7.92	0.32							
13	30	6	3.67	3.19	0.32							
14	30	8	10.72	9.98	0.37							
15	20	19	28.6	27.89	0.26							
16	20	28	16.9	18.7	0.26							
17	40	19	4.62	5.69	0.47							
18	30	2	0.75	0.96	0.34							
19	40	10	6.22	10.52	0.45							
21	30	3	1.22	2.53	0.32							
22	30	20	20.29	39.54	0.39							

DNAcopy Gain

Chromosome Overall (All regions)						Minimal Regions of Interest (p < 0.1)						
Chr	AT 5%	Number CRIs	Mb DNA	Prop. Chr	Max. PWS	Recurrence	Mid Position (Mb)	Mb DNA	GDW	Score	pValue	Genes
7	80	4	38.24	24.03	0.89	0.894736842	54.9851885	0.127725	0	0	0.0000	RP4-791C19; nr. EGFR
						0.815789474	91.92374675	0.3867285	1.571	0.8758	0.0002	PEX1; ERVWE1; GATAD1; KRIT1; CYP51A1; hsa-mir-1285-1; Y_RNA
						0.842105263	27.0406085	7.393609	1.746	0.7956	0.0015	MIR196B; MIR196B; HOTAIRM1; CLK2P; RPS2P32; HOXA11AS; ZNRF2; HOXA4; C7orf30; IGF2BP3; TRA2A; CCDC126; C7orf46; STK31; NPY; MPP6; DFNA5; OSBPL3; CYCS; C7orf31; NPVF; NFE2L3; HNRNPA2B1; CBX3; SNX10; KIAA0087; C7orf71; SKAP2; HOXA1; HOXA2; HOXA3; HOXA5; HOXA6; HOXA7; HOXA9; HOXA10; HOXA11; HOXA13; EVX1; HIBADH; TAX1BP1; JAZF1; CREB5; CPVL; PRR15; WIPF3; SCRN1; FKBP14; PLEKHA8; NOD1; GGCT; GARS; CRHR2; NCRNA00213; CHN2; C7orf41; snoU13; hsa-mir-550-1; U6; Y_RNA; snoU13; SNORD65; snoU13; hsa-mir-196b; U6; hsa-mir-148a; U3
						0.842105263	22.021577	2.272058	2.667	1.1508	0.0024	SP4; DNAH11; CDCA7L; RAPGEF5; IL6; TOMM7; FAM126A; KLHL7; hsa-mir-1183; U1; SRP_euk_arch; SNORD93
4	20	2	7.72	4.04	0.32	0.315789474	54.806086	0.398224	2	2.1076	0.00503	GSX2; CHIC2; RPL21P44; snoU13
7	80	4	38.24	24.03	0.89	0.815789474	15.6302965	2.731487	3	1.1514	0.0078	DGKB; TMEM195; MEOX2; ISPD; SOSTDC1; BZW2; TSPAN13
20	60	2	6.38	10.13	0.66	0.657894737	58.259611	3.147742	1.679	0.6752	0.02099	CTSZ; MTCO2L; C20orf83; MRPS16P; GNASAS; CDH4; C20orf197; CDH26; PPP1R3D; C20orf177; SYCP2; PHACTR3; EDN3; ZNF831; SLMO2; TUBB1; TH1L; GNAS; NPEPL1; STX16; VAPB; RAB22A; PPP4R1L; C20orf85; hsa-mir-296; hsa-mir-646; U7; hsa-mir-298
5	40	3	17.32	9.57	0.47	0.447368421	37.68722325	0.0925875	1.5	0.7116	0.05277	WDR70; U6
5	40	3	17.32	9.57	0.47	0.447368421	38.163874	0.614326	8	3.611	0.06214	EGFLAM
20	60	2	6.38	10.13	0.66	0.605263158	24.248926	1.510958	1.222	0.3926	0.06978	XXyac-YX60D10.1; POM121L3P; CST9L; CST9; CST3; CST4; CST1; CST2; CST5; GGTLA3; C20orf39; CST7; C20orf3; U6; U1; CSTP2; CSTP1; GGTLA3
5	40	3	17.32	9.57	0.47	0.421052632	44.29484925	1.1083685	2.5	1.1124	0.07758	MRPS30; FGF10; SRP_euk_arch; U6
3	40	5	16.64	8.4	0.45	0.447368421	190.2984653	1.3837275	1	0.4061	0.08433	UTS2D; OSTN; IL1RAP; TMEM207; CLDN16; CLDN1; LEPREL1; TP63; 7SK; U6
3	40	5	16.64	8.4	0.45	0.447368421	184.253763	2.245424	1.026	0.3936	0.09739	HSP90AA5P; IGF2BP2; SENP2; LIPH; TMEM41A; MAP3K13; C3orf70; EHHADH; MAGEF1; VPS8; EPHB3; CHRD; THPO; POLR2H; CLCN2; FAM131A; EIF4G1; CAMK2N2; PSMD2; ECE2; VWA5B2; DVL3; EIF2B5; HTR3E; HTR3C; HTR3D; PARL; MAP6D1; YEATS2; KLHL6; KLHL24; MCF2L2; Y_RNA; SNORA63; snoU13; SNORA63; hsa-mir-1224; SNORD66
1	40	2	1.85	0.74	0.45							
2	20	21	64.18	26.39	0.26							
6	30	1	3.44	2.01	0.34							
8	40	3	5.06	3.45	0.45							
9	30	1	0.46	0.33	0.32							
11	30	2	2.43	1.8	0.34							
12	20	9	7.33	5.48	0.26							
13	30	1	7.91	6.87	0.32							
14	30	1	5.58	5.2	0.32							
15	20	8	33.97	33.13	0.29							
16	20	19	18.81	20.82	0.26							
17	40	4	1.32	1.63	0.42							
18	20	1	8.68	11.12	0.29							
19	50	1	1.16	1.96	0.5							
21	20	4	22.27	46.26	0.29							
22	30	5	11.79	22.98	0.39							

GLAD Gain

Chromosome Overall (All regions)						Minimal Regions of Interest (p < 0.1)						
Chr	AT 5%	Number CRIs	Mb DNA	Prop. Chr	Max. PWS	Recurrence	Mid Position (Mb)	Mb DNA	GDW	Score	pValue	Genes
7	80	3	33.01	20.74	0.89	0.894736842	54.897592	0.302918	5	6.5447	#######	SEC61G
						0.815789474	91.92374675	0.3867285	1.571	0.8767	3.05E-04	PEX1; ERVWE1; GATAD1; KRIT1; CYP51A1; hsa-mir-1285-1; Y_RNA
						0.815789474	30.1771505	18.583205	1.74	0.791	2.72E-03	TRGC2; TRGJ2; TRGJP2; TRGJP; TRGJ1; TRGC1; TRGV10; TRGV11; TRGJP1; TRGV9; TRGV5; TRGV8; TRGV4; TRGV3; TRGV2; TRGV1; FAM188B; INMT; TRGV5P; TRGVA; TRGV6; TRGVB; DPY19L2; MIR196B; MIR196B; HOTAIRM1; CLK2P; RPS2P32; HOXA11AS; ZNRF2; HOXA4; SP4; DNAH11; CDCA7L; RAPGEF5; IL6; TOMM7; FAM126A; KLHL7; NUPL2; GPNMB; C7orf30; IGF2BP3; TRA2A; CCDC126; C7orf46; STK31; NPY; MPP6; DFNA5; OSBPL3; CYCS; C7orf31; NPVF; NFE2L3; HNRNPA2B1; CBX3; SNX10; KIAA0087; C7orf71; SKAP2; HOXA1; HOXA2; HOXA3; HOXA5; HOXA6; HOXA7; HOXA9; HOXA10; HOXA11; HOXA13; EVX1; HIBADH; TAX1BP1; JAZF1; CREB5; CPVL; PRR15; WIPF3; SCRN1; FKBP14; PLEKHA8; NOD1; GGCT; GARS; CRHR2; GHRHR; NEUROD6; CCDC129; C7orf16; PDE1C; LSM5; KBTBD2; RP9P; FKBP9; NT5C3; RP9; BBS9; BMPER; NPSR1; DPY19L1; TBX20; HERPUD2; SEPT7; EEPD1; KIAA0895; ELMO1; TXNDC3; SFRP4; STARD3NL; TRGV7; FAM183B; VPS41; POU6F2; NCRNA00213; GPR141; EPDR1; CHN2; C7orf41; U6; SRP_euk_arch; Y_RNA; 5S_rRNA; hsa-mir-1183; U1; SNORA51; SRP_euk_arch; SRP_euk_arch; snoU13; hsa-mir-550-1; U6; Y_RNA; SNORD93; snoU13; SNORD65; snoU13; SNORA20; snoU13; Y_RNA; U6; hsa-mir-196b; U6; U6; hsa-mir-550-2; 5S_rRNA; SRP_euk_arch; hsa-mir-1200; hsa-mir-548n; hsa-mir-148a; U3
4	20	1	2.47	1.29	0.32	0.315789474	54.806086	0.398224	2	2.0555	0.00767	GSX2; CHIC2; RPL21P44; snoU13
20	60	1	3.32	5.27	0.61	0.605263158	58.3471855	3.322891	1.786	0.7643	0.02734	CTSZ; MTCO2L; C20orf83; MRPS16P; GNASAS; CDH4; C20orf197; CDH26; PPP1R3D; C20orf177; SYCP2; PHACTR3; EDN3; ZNF831; SLMO2; TUBB1; TH1L; GNAS; NPEPL1; STX16; VAPB; RAB22A; PPP4R1L; C20orf85; hsa-mir-296; hsa-mir-646; U7; hsa-mir-298
5	40	3	33.13	18.31	0.45	0.447368421	32.165986	0.03893	3	1.5493	0.044	GOLPH3
1	30	10	104.63	41.98	0.39	0.394736842	200.968753	1.073916	1.444	0.7885	0.05631	RPS10P7; RPL34P1; CSRP1; TNNI1; PHLDA3; LAD1; PKP1; TNNT2; IGFN1; TMEM9; CACNA1S; KIF21B; C1orf106; GPR25; CAMSAP1L1; KIF14; DDX59; U6
5	40	3	33.13	18.31	0.45	0.447368421	37.89945675	1.1431605	2.5	1.1091	0.07271	EGFLAM; GDNF; WDR70; NUP155; U6; U6
5	40	3	33.13	18.31	0.45	0.421052632	44.7109645	1.940599	2.8	1.2205	0.08992	HCN1; MRPS30; FGF10; SRP_euk_arch; U6
19	40	1	2.17	3.66	0.45	0.447368421	36.908719	0.436184	0.375	0.1482	0.09865	ZNF382; ZNF529; ZNF566; ZFP82; ZFP14; ZNF146; ZNF565; Y_RNA
2	20	5	26.63	10.95	0.24							
3	40	5	15.79	7.97	0.45							
6	20	7	48.58	28.39	0.26							
8	40	1	0.58	0.4	0.42							
9	20	5	23.48	16.63	0.24							
11	30	1	0.45	0.33	0.32							
12	20	2	0.68	0.51	0.21							
13	20	2	91.49	79.44	0.29							
14	20	2	59.37	55.31	0.26							
15	20	2	34.45	33.6	0.24							
16	20	9	8.92	9.87	0.24							
17	30	2	38.32	47.19	0.39							
18	20	1	8.65	11.08	0.26							
21	30	1	0.13	0.27	0.32							
22	30	2	8.5	16.57	0.32							

Homogeneous HMM Gain

Chr	AT 5%	Number CRIs	Mb DNA	Prop. Chr	Max. PWS	Recurrence	Mid Position (Mb)	Mb DNA	GDW	Score	pValue	Genes
Chromosome Overall (All regions)						Minimal Regions of Interest (p < 0.1)						
7	80	14	17.82	11.2	0.89	0.894736842	54.872287	0.252308	4	4.3118	3.22E-15	SEC61G
					0.89	0.815789474	91.90800975	0.3552545	2	1.1621	7.49E-05	GATAD1; KRIT1; CYP51A1; hsa-mir-1285-1; Y RNA
					0.89	0.815789474	26.97294675	0.0750125	11	5.5077	0.001	SKAP2
					0.89	0.815789474	27.7386355	1.455107	0.833	0.4023	0.001	MIR196B; MIR196B; HOTAIRM1; HOXA11AS; HOXA4; SKAP2; HOXA1; HOXA2; HOXA3; HOXA5; HOXA6; HOXA7; HOXA9; HOXA10; HOXA11; HOXA13; EVX1; HIBADH; TAX1BP1; JAZF1; CREB5; NCRNA00213; hsa-mir-196b; U6
					0.89	0.815789474	30.1537635	0.653069	1.778	0.838	0.001	ZNRF2; WIPF3; SCRN1; FKBP14; PLEKHA8; NOD1; C7orf41; hsa-mir-550-1; snoU13
					0.89	0.815789474	33.0358835	0.686869	1.333	0.6238	0.001	KBTBD2; RP9P; FKBP9; NT5C3; RP9; BBS9; 5S rRNA; SRP euk arch; hsa-mir-550-2
					0.89	0.815789474	46.968329	2.867128	2.4	1.1014	0.001	EPS15L2; SEPT13; IGFBP1; IGFBP3; TNS3; C7orf65; HUS1; PKD1L1; C7orf69; SUNC1; C7orf57; UPP1; U6; U7; U6
					0.89	0.815789474	38.63389175	1.2872775	0.571	0.2589	0.002	TRGC2; TRGJ2; TRGJP2; TRGJP; TRGJ1; TRGC1; TRGV10; TRGV11; TRGJP1; TRGV9; TRGV5; TRGV8; TRGV4; TRGV3; TRGV2; TRGV1; TRGV5P; TRGVA; TRGV6; TRGVB; SFRP4; STARD3NL; TRGV7; FAM183B; VPS41; POU6F2; EPDR1; SRP euk arch
					0.89	0.815789474	24.244373	1.812674	1.529	0.6925	0.002	CLK2P; RPS2P32; C7orf30; IGF2BP3; TRA2A; CCDC126; C7orf46; STK31; NPY; MPP6; DFNA5; OSBPL3; snoU13; U6; Y RNA; snoU13; SNORD65
					0.89	0.815789474	21.9276225	2.526969	2.615	1.1833	0.002	SP8; SP4; DNAH11; CDCA7L; RAPGEF5; IL6; TOMM7; FAM126A; KLHL7; hsa-mir-1183; U1; SRP_euk_arch; SNORD93
					0.89	0.815789474	44.3963415	0.527537	1.455	0.6501	0.002	POLD2; MYL7; GCK; YKT6; CAMK2B; NUDCD3; NPC1L1; DDX56; TMED4; OGDH; U6
					0.89	0.815789474	36.3594515	0.254715	2	0.8696	0.002	EEPD1; KIAA0895; Y_RNA
					0.89	0.815789474	42.6276285	2.615425	3.077	1.3322	0.003	INHBA; GLI3; PSMA2; C7orf25; MRPL32; HECW1; STK17A; C7orf44; BLVRA; MRPS24; URG4; Y RNA; U6
					0.89	0.815789474	35.577834	1.032726	2.5	1.0478	0.003	DPY19L2; DPY19L1; TBX20; HERPUD2; SEPT7; U6
4	20	15	8.91	4.66	0.34	0.342105263	54.806086	0.398224	2	1.9598	0.004	GSX2; CHIC2; RPL21P44; snoU13
20	60	17	12.04	19.1	0.68	0.684210526	44.6253755	0.120483	0.8	0.364	0.00688	FTLP; PCIF1; ZNF335; MMP9; SLC12A5
					0.68	0.684210526	56.715494	0.103328	5	2.2462	0.00752	C20orf85
4	20	15	8.91	4.66	0.34	0.342105263	55.3488785	0.197025	0	0	0.00991	NA
20	60	17	12.04	19.1	0.68	0.684210526	57.0270185	0.036269	4	1.6609	0.0125	VAPB
					0.68	0.631578947	46.516291	0.415958	1.5	0.6681	0.01326	SRMP1; SULF2; U7; U7
					0.68	0.684210526	57.6522155	0.586247	0.909	0.3557	0.01754	CTSZ; MRPS16P; GNASAS; EDN3; ZNF831; SLMO2; TUBB1; TH1L; GNAS; hsa-mir-296; hsa-mir-298
					0.68	0.631578947	40.9166945	0.679733	3	1.2711	0.01758	PTPRT
					0.68	0.605263158	36.675193	0.094976	2	0.8769	0.01837	KIAA0406; RPRD1B
					0.68	0.605263158	60.9074455	0.275059	1.5	0.6412	0.02096	GATA5; C20orf151; CABLES2; RPS21; LAMA5; OSBPL2; HRH3; GTPBP5
					0.68	0.605263158	42.0744925	0.133331	0.667	0.2843	0.02121	EIF4EBP2P; SFRS6; L3MBTL; U6; Y RNA; U6
					0.68	0.605263158	30.34283725	0.2919315	1	0.4255	0.02146	COX4I2; BCL2L1; TPX2; MYLK2; FOXS1; DUSP15; TTLL9
					0.68	0.684210526	58.388277	0.622834	0.857	0.3184	0.02293	C20orf197; CDH26; PPP1R3D; C20orf177; SYCP2; PHACTR3; U7
					0.68	0.631578947	38.5255555	0.568367	6	2.3968	0.02376	HSPEP1
					0.68	0.631578947	37.347778	0.480086	0.625	0.2417	0.0277	RPS3P2; KIAA1219; C20orf95; SLC32A1; PPP1R16B; FAM83D; SRP_euk_arch; 7SK
					0.68	0.631578947	44.155516	0.153866	0.857	0.3265	0.02965	WFDC6; RPL5P2; SPINLW1; WFDC2; SPINT3; WFDC8; 5S_rRNA
					0.68	0.605263158	42.5956615	0.065577	3	1.1675	0.03252	C20orf100
					0.68	0.605263158	29.7962755	0.069531	0	0	0.03298	NA
					0.68	0.605263158	31.6437205	0.030187	1.5	0.5186	0.0515	BPIL3; C20orf185
					0.68	0.605263158	29.40354	0.102744	0	0	0.06124	NA
					0.68	0.605263158	9.076532	0.765236	3.333	1.0862	0.06274	PLCB4; PLCB1; SNORA73
17	40	7	0.52	0.64	0.47	0.473684211	41.5955355	0.057931	1.5	0.6036	0.06967	ETV4; DHX8
20	60	17	12.04	19.1	0.68	0.605263158	23.8622755	0.799789	0.6	0.1873	0.07147	XXyac-YX60D10.1; POM121L3P; CST8; CST9L; CST9; CST3; CST4; CST1; CST2; CST5; GGTLA3; U1; CSTP2; CSTP1; GGTLA3
8	40	13	11.95	8.17	0.47	0.473684211	121.5689355	0.070337	1.5	0.5924	0.07361	MTBP; SNTB1
					0.47	0.473684211	124.874236	0.506656	3.333	1.275	0.08057	KLHL38; FAM91A1; FER1L6
5	50	5	4.76	2.63	0.53	0.5	9.174911	0.1608	3	1.0799	0.08204	SEMA5A
17	40	7	0.52	0.64	0.47	0.421052632	74.47967	0.035492	3	1.2443	0.08886	RHBDF2
					0.47	0.421052632	76.7146585	0.1118125	1.5	0.6126	0.09244	USP36; CYTH1
8	40	13	11.95	8.17	0.47	0.447368421	118.1390945	0.382167	2.667	1.0127	0.09525	C8orf85; SLC30A8; SRP_euk_arch
3	40	8	5.86	2.96	0.45	0.447368421	190.6002995	0.463407	4	1.5059	0.09729	IL1RAP
1	30	38	17.2	6.9	0.37							
2	30	12	4.93	2.03	0.34							
6	30	20	8.83	5.16	0.34							
9	30	6	1.67	1.18	0.34							
11	30	11	6.68	4.95	0.37							
12	20	24	10.45	7.81	0.32							
13	30	8	5.85	5.08	0.32							
14	30	16	12.79	11.92	0.37							
15	30	1	0.14	0.13	0.32							
16	30	3	0.64	0.71	0.32							
18	30	4	2.64	3.39	0.34							
19	40	10	6.21	10.5	0.47							
21	30	6	7.35	15.28	0.39							
22	30	37	10.56	20.59	0.39							

Supplementary Table S1B (Copy number loss data)

Biologically Tuned HMM Loss

Chromosome Overall (All regions)						Minimal Regions of Interest (p < 0.1)						
**Chr**	**AT 5%**	**Number CRIs**	**Mb DNA**	**Prop. Chr**	**Max. PWS**	**Recurrence**	**id Position (M**	**Mb DNA**	**GDW**	**Score**	**pValue**	**Genes**
9	60	1	7.46	5.29	0.89	0.894736842	21.8658185	0.264757	1.5	-2.129226053	2.90E-024	CDKN2BAS; MTAP; C9orf53; CDKN2A
						0.842105263	26.425852	0.147118	3	-1.31318991	0.0015	MYO3A
						0.815789474	12.7528015	0.197845	1.5	-0.67338708	0.0016	CAMK1D; MIR548Q
						0.815789474	26.9705255	0.321065	2	-0.887355528	0.0017	C10orf50; PDSS1; SNORA57; U6
						0.815789474	24.15062425	1.6267945	1.272727273	-0.563471414	0.0018	PRINS; MSRB2; PTF1A; C10orf115; C10orf67; OTUD1; KIAA1217; snoU13; 5S_rRNA; U4; hsa-mir-603
						0.815789474	28.253949	0.124994	0	0	0.0018	NA
						0.842105263	25.399507	0.438356	1.25	-0.529876697	0.0020	PRTFDC1; ENKUR; THNSL1; GPR158
						0.815789474	12.3471535	0.190893	2.5	-1.084455076	0.0021	CAMK1D; CDC123
						0.815789474	29.10892	0.201274	1.666666667	-0.719401032	0.0022	C10orf126; U6; 5S_rRNA
10	80	37	18.96	13.99	0.87	0.815789474	12.008937	0.146134	4	-1.662095424	0.0031	UPF2
						0.815789474	16.507721	0.333412	1	-0.415337469	0.0031	PTER; C1QL1; RSU1; snoU13; U2
						0.842105263	11.5507215	0.278669	3	-1.202262421	0.0032	USP6NL
						0.842105263	9.441817	2.371964	5.5	-2.184596896	0.0034	Y_RNA; 5S_rRNA
						0.815789474	19.6262475	0.813461	2.5	-1.019593252	0.0036	C10orf112; U3
						0.815789474	0.7770605	0.519637	0.75	-0.305714839	0.0036	GTPBP4; LARP4B; C10orf108; DIP2C
						0.868421053	133.6221105	0.044281	0	0	0.0037	NA
						0.842105263	1.20367175	0.2389245	1	-0.390045691	0.0039	C10orf139; C10orf110; WDR37; IDI1; U7
						0.842105263	2.1836455	0.447867	3	-1.164642629	0.0041	U6
						0.868421053	87.7201785	0.323873	2.5	-0.936126422	0.0043	GRID1; 5S_rRNA
11	40	1	0.15	0.11	0.42	0.421052632	113.2765165	0.153955	3	-1.420764292	0.0508	DRD2; TTC12
14	60	4	0.27	0.25	0.61	0.605263158	24.06394125	0.1662035	1.25	-0.38510965	0.0624	DHRS2; JPH4; THTPA; ZFHX2
4	40	18	10.98	5.74	0.47	0.473684211	92.493808	0.752692	2	-0.761592254	0.0685	FAM190A; 5S_rRNA
14	60	4	0.27	0.25	0.61	0.605263158	38.7956555	0.045137	0	0	0.0883	NA
13	70	2	0.39	0.34	0.74	0.736842105	18.7022315	0.200997	0	0	0.0914	NA
1	30	21	11.28	4.53	0.42							
2	30	17	5.94	2.44	0.42							
3	30	27	9.46	4.78	0.45							
5	30	5	0.86	0.48	0.32							
6	30	10	8.82	5.15	0.42							
7	20	2	1.04	0.65	0.24							
8	30	8	3.01	2.06	0.37							
12	50	4	1.88	1.4	0.55							
15	30	13	7.16	6.99	0.39							
16	40	3	0.4	0.44	0.53							
17	30	18	2.72	3.35	0.37							
18	40	1	0.26	0.34	0.42							
19	30	14	4.17	7.04	0.39							
20	20	13	3.24	5.14	0.29							
21	40	1	3.47	7.21	0.45							
22	40	7	2.45	4.77	0.45							

DNAcopy Loss

Chromosome Overall (All regions)						Minimal Regions of Interest (p < 0.1)						
Chr	AT 5%	Number CRIs	Mb DNA	Prop. Chr	Max. PWS	Recurrence	Mid Position (M	Mb DNA	GDW	Score	pValue	Genes
9	60	1	7.46	5.285	0.89	0.894736842	21.8658185	0.264757	1.5	-2.644066176	1.86E-036	CDKN2BAS; MTAP; C9orf53; CDKN2A
10	70	8	75.54	55.738	0.79	0.789473684	24.877968	8.663178	1.682539683	-0.752213333	0.002129976	C10orf52; C10orf51; C10orf50; PRINS; PLXDC2; NEBL; C10orf113; C10orf114; C10orf140; MLLT10; DNAJC1; COMMD3; BMI1; SPAG6; PIP5K2A; MSRB2; PTF1A; C10orf115; C10orf67; OTUD1; KIAA1217; PRTFDC1; ENKUR; THNSL1; GPR158; MYO3A; GAD2; PDSS1; YME1L1; MASTL; PTCHD3; RAB18; MKX; MPP7; WAC; BAMBI; C10orf126; snoU13; U6; 5S_rRNA; U4; 5S_rRNA; 7SK; U6; U6; Y_RNA; U6; 5S_rRNA; SNORA57; 7SK; hsa-mir-603; U6; U6; snoU13; U6; 5S_rRNA; hsa-mir-1915; 7SK; U6; 5S_rRNA; U3; U6; U6
						0.789473684	11.1589275	1.062257	2.5	-1.071061667	0.003056652	SFTA1P; USP6NL; C10orf31; CUGBP2
1	30	2	8.88	3.564	0.39	0.342105263	51.275515	0.3891	3	-2.819550306	0.004535859	CDKN2C; FAF1
						0.789473684	87.624437	0.515356	3	-1.21304	0.004783922	GRID1; 5S_rRNA
						0.763157895	38.4245825	0.515423	1.6	-0.635635862	0.006869475	ZNF25; ZNF33A; ZNF37A; HSD17B7P2; Y_RNA
						0.763157895	133.506942	0.92377	2.5	-0.946531034	0.009356091	BNIP3; JAKMIP3; PPP2R2D; TCERG1L
10	70	8	75.54	55.738	0.79	0.763157895	123.1758245	1.715085	2.6	-0.98318069	0.009427163	RPS15AP5; TACC2; NSMCE4A; FGFR2; C10orf85; BRWD2; PPAPDC1A; BTBD16; Y_RNA; 7SK
						0.763157895	128.053652	0.532908	3	-1.122027586	0.010079137	C10orf90; 5S_rRNA
						0.710526316	108.0894635	4.080661	2.777777778	-1.052140741	0.014108302	SORCS1; SORCS3; CCDC147; ITPRIP; GSTO2; U6; 5S_rRNA; snoU13; 5S_rRNA
						0.710526316	49.654245	0.748988	3.75	-1.309372222	0.021298479	FRMPD2; MAPK8; WDFY4; PTPN20C
						0.710526316	42.5923505	0.605099	3	-1.033853086	0.022640101	BX322613.2; BX322613.1; BX322613.3
						0.710526316	79.636228	0.609036	0.833333333	-0.22241358	0.058837071	RPS24; POLR3A; DLG5; KCNMA1; 5S_rRNA; U6
11	30	6	10.47	7.758	0.39	0.315789474	107.5300975	0.820415	4	-2.177119444	0.077232038	CUL5; RAB39; SLC35F2; SLN; ELMOD1; CWF19L2
13	50	9	40.91	35.525	0.58	0.578947368	49.357849	0.369072	4.5	-1.334384091	0.077464724	CYSLTR2; 5S_rRNA
11	30	6	10.47	7.758	0.39	0.315789474	104.29522	1.14011	2.4	-1.265186667	0.083621339	CASP5; CASP4; CASP12; DDI1; PDGFD
						0.315789474	109.521102	0.626452	8	-4.163816667	0.086221096	C11orf87
14	50	4	12.53	11.677	0.58	0.578947368	26.884892	4.58592	0.815789474	-0.220784928	0.096184539	NOVA1; STXBP6; GZMB; GZMH; CTSG; CMA1; SDR39U1; KHNYN; CBLN3; NYNRIN; NFATC4; RIPK3; LTB4R; LTB4R2; CIDEB; C14orf21; DHRS1; RABGGTA; TGM1; TINF2; GMPR2; NEDD8; MDP1; CHMP4A; TSSK4; TM9SF1; IPO4; REC8; IRF9; RNF31; PSME2; FAM158A; PSME1; FITM1; WDR23; SNORD37; 5S_rRNA; U6
11	30	6	10.47	7.758	0.39	0.342105263	116.3950173	0.3224795	0	0	0.097311863	NA
2	30	1	0.64	0.264	0.32							
3	30	4	2.82	1.425	0.34							
4	30	11	90.2	47.188	0.39							
5	20	13	20.95	11.578	0.26							
6	30	1	0.15	0.088	0.37							
7	20	1	0.29	0.179	0.21							
8	30	3	7.99	5.457	0.34							
12	30	11	50.13	37.453	0.39							
15	30	3	5.65	5.51	0.37							
16	30	9	9.91	10.973	0.34							
17	20	11	17.48	21.53	0.29							
18	30	8	31.57	40.437	0.39							
19	20	1	23.9	40.421	0.29							
20	20	1	23.9	40.421	0.29							
21	20	4	16.85	35.004	0.29							
22	30	7	8.07	15.731	0.34							

GLAD Loss

Chromosome Overall (All regions)						Minimal Regions of Interest (p < 0.1)						
**Chr**	**AT 5%**	**Number CRIs**	**Mb DNA**	**Prop. Chr**	**Max. PWS**	**Recurrence**	**id Position (M**	**Mb DNA**	**GDW**	**Score**	**pValue**	**Genes**
9	60	1	5.99	4.241	0.89	0.894736842	21.8658185	0.264757	1.5	-3.02311909	2.46E-044	CDKN2BAS; MTAP; C9orf53; CDKN2A
1	30	3	9.05	3.629	0.39	0.342105263	51.275515	0.3891	3	-3.303320705	0.001530432	CDKN2C; FAF1
						0.710526316	87.624437	0.515356	3	-1.348667283	0.005839841	GRID1; 5S_rRNA
						0.763157895	128.053652	0.532908	3	-1.142848307	0.010688024	C10orf90; 5S_rRNA
10	70	3	61.57	45.425	0.79	0.789473684	38.5794035	0.222759	3	-1.051896325	0.014101773	HSD17B7P2
14	50	2	10.45	9.731	0.58	0.578947368	24.0629455	0.168195	1.5	-0.411010775	0.099085233	DHRS2; JPH4; THTPA; ZFHX2
13	50	7	39.62	34.4	0.53	0.526315789	50.575241	3.169932	1.540540541	-0.46370302	0.099372829	DLEU7; COX7CP1; DLEU2; FAM10A4; SERPINE3; WDFY2; INTS6; FAM124A; GUCY1B2; RNASEH2B; DLEU1; KCNRG; TRIM13; C13orf1; KPNA3; EBPL; RCBTB1; PHF11; SETDB2; CAB39L; CDADC1; MLNR; FNDC3A; CYSLTR2; RCBTB2; LPAR6; RB1; SRP_euk_arch; hsa-mir-16-1; 5S_rRNA; 5S_rRNA; 5S_rRNA; Y_RNA; hsa-mir-15a; Y_RNA; U6; Y_RNA
2	30	2	1.16	0.476	0.34							
3	30	1	0.73	0.37	0.32							
4	30	6	39.92	20.883	0.37							
5	20	7	14.58	8.062	0.24							
6	30	1	0.15	0.088	0.32							
8	20	2	44.51	30.411	0.29							
11	30	2	5.64	4.174	0.34							
12	30	5	18.76	14.013	0.34							
15	30	2	1.86	1.819	0.34							
16	20	2	35.29	39.052	0.29							
17	20	7	3.44	4.242	0.26							
18	30	6	25.39	32.518	0.34							
19	20	1	23.9	40.421	0.26							
21	30	1	3.82	7.934	0.37							
22	30	4	4.73	9.222	0.34							

Homogeneous HMM Loss

Chromosome Overall (All regions)						Minimal Regions of Interest (p < 0.1)						
**Chr**	**AT 5%**	**Number CRIs**	**Mb DNA**	**Prop. Chr**	**Max. PWS**	**Recurrence**	**id Position (M**	**Mb DNA**	**GDW**	**Score**	**pValue**	**Genes**
9	50	2	9.67	6.84	0.68	0.684210526	22.0808515	1.218563	1.375	-1.700426409	1.43E-012	CDKN2BAS; MTAP; IFNE; C9orf53; CDKN2A; CDKN2B; DMRTA1; hsa-mir-31
						0.815789474	88.609891	0.393474	0.6	-0.242373989	0.002824962	FAM25A; C10orf116; SNCG; MMRN2; BMPR1A; LDB3; OPN4; BMS1P3; U6; U1
10	80	3	1.02	0.75	0.82	0.815789474	130.0023575	0.407323	2.5	-0.974098998	0.003768875	MKI67; PTPRE
						0.815789474	98.9062815	0.219007	5	-1.696248759	0.009730665	SLIT1
11	30	28	17.92	13.27	0.39	0.368421053	107.814429	0.105122	2.5	-1.405672999	0.038493176	RAB39; SLC35F2
14	60	11	4.27	3.97	0.68	0.657894737	24.095536	0.100012	1.5	-0.46989524	0.039219562	DHRS2; JPH4
11	30	28	17.92	13.27	0.39	0.315789474	109.507784	0.599816	7	-4.165729502	0.053531675	C11orf87
14	60	11	4.27	3.97	0.68	0.631578947	27.5522795	1.365137	10	-2.860282792	0.060315826	NOVA1
11	30	28	17.92	13.27	0.39	0.315789474	104.2106395	1.309271	2.6	-1.464398825	0.063085413	CASP5; CASP4; CASP12; DDI1; PDGFD
14	60	11	4.27	3.97	0.68	0.684210526	38.7609815	0.088249	5	-1.297678545	0.063374245	CLEC14A
13	60	9	2.81	2.44	0.63	0.631578947	26.4066385	0.424403	2.5	-0.688619756	0.067120556	TMEM46; Y_RNA
14	60	11	4.27	3.97	0.68	0.605263158	26.06211	0.153492	0	0	0.074460502	NA
13	60	9	2.81	2.44	0.63	0.605263158	50.975263	0.314958	6	-1.641197221	0.076507979	DLEU1
9	50	2	9.67	6.84	0.68	0.5	31.619394	0.524102	0	0	0.078481853	NA
11	30	28	17.92	13.27	0.39	0.368421053	113.113998	0.171082	2.5	-1.109131037	0.079011892	TTC12; NCAM1
14	60	11	4.27	3.97	0.68	0.605263158	23.8316125	0.044125	1	-0.269524858	0.079394161	MYH6; CMTM5; IL25; EFS; SLC22A17
14	60	11	4.27	3.97	0.68	0.605263158	20.92809625	0.1734885	0.857142857	-0.222568706	0.086845303	RNASE10; NP; TMEM55B; OSGEP; KLHL33; TEP1; 5S_rRNA
13	60	9	2.81	2.44	0.63	0.605263158	26.81138375	0.1727815	2	-0.510241573	0.090448529	RNF6; CDK8
13	60	9	2.81	2.44	0.63	0.605263158	26.106365	0.10441	5	-1.25234739	0.094251715	U6
14	60	11	4.27	3.97	0.68	0.605263158	23.71335875	0.0316115	7	-1.748792132	0.094785808	C14orf164
11	30	28	17.92	13.27	0.39	0.368421053	116.622076	0.131638	2	-0.804858313	0.09891034	ZNF259; BUD13
1	30	6	1.03	0.41	0.34							
2	30	32	12.51	5.14	0.53							
3	30	20	9.5	4.8	0.39							
4	40	5	3	1.57	0.47							
5	30	12	2.28	1.26	0.39							
6	40	4	0.7	0.41	0.53							
7	20	7	2.97	1.87	0.26							
8	40	3	1.71	1.17	0.45							
12	40	22	9.99	7.46	0.5							
15	40	4	2.12	2.07	0.45							
16	40	12	5.13	5.67	0.66							
17	30	10	2.26	2.78	0.39							
18	50	3	1.23	1.58	0.55							
19	30	15	2.83	4.78	0.5							
20	30	2	0.34	0.53	0.42							
21	50	1	3.47	7.21	0.58							
22	40	5	0.86	1.67	0.45							

Supplementary Table S2. Prioritization by filtering on amplitude (p<0.1), then by modified GTS

DNAcopy Gain

Chr	Recurrence	Mid Position (Mb)	Reg Size	GDW	Score	pValue	Genes
5	0.447368421	38.163874	0.614326	8	3.611	0.06213861	EGFLAM
4	0.315789474	54.806086	0.398224	2	2.1076	0.00503348	GSX2; CHIC2; RPL21P44; snoU13
7	0.815789474	15.6302965	2.731487	3	1.1514	0.0078	DGKB; TMEM195; MEOX2; ISPD; SOSTDC1; BZW2; TSPAN13
7	0.842105263	22.021577	2.272058	2.667	1.1508	0.0024	SP4; DNAH11; CDCA7L; RAPGEF5; IL6; TOMM7; FAM126A; KLHL7; hsa-mir-1183; U1; SRP_euk_arch; SNORD93
5	0.421052632	44.29484925	1.1083685	2.5	1.1124	0.07758147	MRPS30; FGF10; SRP_euk_arch; U6
7	0.815789474	91.92374675	0.3867285	1.571	0.8758	0.0002	PEX1; ERVWE1; GATAD1; KRIT1; CYP51A1; hsa-mir-1285-1; Y_RNA
7	0.842105263	27.0406085	7.393609	1.746	0.7956	0.0015	MIR196B; MIR196B; HOTAIRM1; CLK2P; RPS2P32; HOXA11AS; ZNRF2; HOXA4; C7orf30; IGF2BP3; TRA2A; CCDC126; C7orf46; STK31; NPY; MPP6; DFNA5; OSBPL3; CYCS; C7orf31; NPVF; NFE2L3; HNRNPA2B1; CBX3; SNX10; KIAA0087; C7orf71; SKAP2; HOXA1; HOXA2; HOXA3; HOXA5; HOXA6; HOXA7; HOXA9; HOXA10; HOXA11; HOXA13; EVX1; HIBADH; TAX1BP1; JAZF1; CREB5; CPVL; PRR15; WIPF3; SCRN1; FKBP14; PLEKHA8; NOD1; GGCT; GARS; CRHR2; NCRNA00213; CHN2; C7orf41; snoU13; hsa-mir-550-1; U6; Y_RNA; snoU13; SNORD65; snoU13; hsa-mir-196b; U6; hsa-mir-148a; U3
5	0.447368421	37.68722325	0.0925875	1.5	0.7116	0.05277212	WDR70; U6
20	0.657894737	58.259611	3.147742	1.679	0.6752	0.02098746	CTSZ; MTCO2L; C20orf83; MRPS16P; GNASAS; CDH4; C20orf197; CDH26; PPP1R3D; C20orf177; SYCP2; PHACTR3; EDN3; ZNF831; SLMO2; TUBB1; TH1L; GNAS; NPEPL1; STX16; VAPB; RAB22A; PPP4R1L; C20orf85; hsa-mir-296; hsa-mir-646; U7; hsa-mir-298
3	0.447368421	190.2984653	1.3837275	1	0.4061	0.08433351	UTS2D; OSTN; IL1RAP; TMEM207; CLDN16; CLDN1; LEPREL1; TP63; 7SK; U6
3	0.447368421	184.253763	2.245424	1.026	0.3936	0.09738826	HSP90AA5P; IGF2BP2; SENP2; LIPH; TMEM41A; MAP3K13; C3orf70; EHHADH; MAGEF1; VPS8; EPHB3; CHRD; THPO; POLR2H; CLCN2; FAM131A; EIF4G1; CAMK2N2; PSMD2; ECE2; VWA5B2; DVL3; EIF2B5; HTR3E; HTR3C; HTR3D; PARL; MAP6D1; YEATS2; KLHL6; KLHL24; MCF2L2; Y_RNA; SNORA63; snoU13; SNORA63; hsa-mir-1224; SNORD66
20	0.605263158	24.248926	1.510958	1.222	0.3926	0.06978204	XXyac-YX60D10.1; POM121L3P; CST9L; CST9; CST3; CST4; CST1; CST2; CST5; GGTLA3; C20orf39; CST7; C20orf3; U6; U1; CSTP2; CSTP1; GGTLA3
7	0.894736842	54.9851885	0.127725	0	0	0.0000	RP4-791C19; nr. EGFR

DNAcopy Loss

Chr	Recurrence	Mid Position (Mb)	Reg Size	GDW	Score	pValue	Genes
11	0.315789474	109.521102	0.626452	8	-4.164	0.0862211	C11orf87
1	0.342105263	51.275515	0.3891	3	-2.82	0.00453586	CDKN2C; FAF1
9	0.894736842	21.8658185	0.264757	1.5	-2.644	1.86E-036	CDKN2BAS; MTAP; C9orf53; CDKN2A
11	0.315789474	107.5300975	0.820415	4	-2.177	0.07723204	CUL5; RAB39; SLC35F2; SLN; ELMOD1; CWF19L2
13	0.578947368	49.357849	0.369072	4.5	-1.334	0.07746472	CYSLTR2; 5S_rRNA
10	0.710526316	49.654245	0.748988	3.75	-1.309	0.02129848	FRMPD2; MAPK8; WDFY4; PTPN20C
11	0.315789474	104.29522	1.14011	2.4	-1.265	0.08362134	CASP5; CASP4; CASP12; DDI1; PDGFD
10	0.789473684	87.624437	0.515356	3	-1.213	0.00478392	GRID1; 5S_rRNA
10	0.763157895	128.053652	0.532908	3	-1.122	0.01007914	C10orf90; 5S_rRNA
10	0.789473684	11.1589275	1.062257	2.5	-1.071	0.00305665	SFTA1P; USP6NL; C10orf31; CUGBP2
10	0.710526316	108.0894635	4.080661	2.778	-1.052	0.0141083	SORCS1; SORCS3; CCDC147; ITPRIP; GSTO2; U6; 5S_rRNA; snoU13; 5S_rRNA
10	0.710526316	42.5923505	0.605099	3	-1.034	0.0226401	BX322613.2; BX322613.1; BX322613.3
10	0.763157895	123.1758245	1.715085	2.6	-0.983	0.00942716	RPS15AP5; TACC2; NSMCE4A; FGFR2; C10orf85; BRWD2; PPAPDC1A; BTBD16; Y_RNA; 7SK
10	0.763157895	133.506942	0.92377	2.5	-0.947	0.00935609	BNIP3; JAKMIP3; PPP2R2D; TCERG1L
10	0.789473684	24.877968	8.663178	1.683	-0.752	0.00212998	C10orf52; C10orf51; C10orf50; PRINS; PLXDC2; NEBL; C10orf113; C10orf114; C10orf140; MLLT10; DNAJC1; COMMD3; BMI1; SPAG6; PIP5K2A; MSRB2; PTF1A; C10orf115; C10orf67; OTUD1; KIAA1217; PRTFDC1; ENKUR; THNSL1; GPR158; MYO3A; GAD2; PDSS1; YME1L1; MASTL; PTCHD3; RAB18; MKX; MPP7; WAC; BAMBI; C10orf126; snoU13; U6; 5S_rRNA; U4; 5S_rRNA; 7SK; U6; U6; Y_RNA; U6; 5S_rRNA; SNORA57; 7SK; hsa-mir-603; U6; U6; snoU13; U6; 5S_rRNA; hsa-mir-1915; 7SK; U6; 5S_rRNA; U3; U6; U6
10	0.763157895	38.4245825	0.515423	1.6	-0.636	0.00686948	ZNF25; ZNF33A; ZNF37A; HSD17B7P2; Y_RNA
10	0.710526316	79.636228	0.609036	0.833	-0.222	0.05883707	RPS24; POLR3A; DLG5; KCNMA1; 5S_rRNA; U6
14	0.578947368	26.884892	4.58592	0.816	-0.221	0.09618454	NOVA1; STXBP6; GZMB; GZMH; CTSG; CMA1; SDR39U1; KHNYN; CBLN3; NYNRIN; NFATC4; RIPK3; LTB4R; LTB4R2; CIDEB; C14orf21; DHRS1; RABGGTA; TGM1; TINF2; GMPR2; NEDD8; MDP1; CHMP4A; TSSK4; TM9SF1; IPO4; REC8; IRF9; RNF31; PSME2; FAM158A; PSME1; FITM1; WDR23; SNORD37; 5S_rRNA; U6
11	0.342105263	116.3950173	0.3224795	0	0	0.09731186	NA

Supplementary Table S2. Prioritization by filtering on amplitude (p<0.1), then by modified GTS

GLAD Gains

Chr	Recurrence	Mid Position (Mb)	Reg Size	GDW	Score	pValue	Genes
7	0.894736842	54.897592	0.302918	5	6.5447	0.00E+00	SEC61G
4	0.315789474	54.806086	0.398224	2	2.0555	0.00766976	GSX2; CHIC2; RPL21P44; snoU13
5	0.447368421	32.165986	0.03893	3	1.5493	0.0440033	GOLPH3
5	0.421052632	44.7109645	1.940599	2.8	1.2205	0.08992082	HCN1; MRPS30; FGF10; SRP_euk_arch; U6
5	0.447368421	37.89945675	1.1431605	2.5	1.1091	0.07270708	EGFLAM; GDNF; WDR70; NUP155; U6; U6
7	0.815789474	91.92374675	0.3867285	1.571	0.8767	3.05E-04	PEX1; ERVWE1; GATAD1; KRIT1; CYP51A1; hsa-mir-1285-1; Y_RNA
7	0.815789474	30.1771505	18.583205	1.74	0.791	2.72E-03	TRGC2; TRGJ2; TRGJP2; TRGJP; TRGJ1; TRGC1; TRGV10; TRGV11; TRGJP1; TRGV9; TRGV5; TRGV8; TRGV4; TRGV3; TRGV2; TRGV1; FAM188B; INMT; TRGV5P; TRGVA; TRGV6; TRGVB; DPY19L2; MIR196B; MIR196B; HOTAIRM1; CLK2P; RPS2P32; HOXA11AS; ZNRF2; HOXA4; SP4; DNAH11; CDCA7L; RAPGEF5; IL6; TOMM7; FAM126A; KLHL7; NUPL2; GPNMB; C7orf30; IGF2BP3; TRA2A; CCDC126; C7orf46; STK31; NPY; MPP6; DFNA5; OSBPL3; CYCS; C7orf31; NPVF; NFE2L3; HNRNPA2B1; CBX3; SNX10; KIAA0087; C7orf71; SKAP2; HOXA1; HOXA2; HOXA3; HOXA5; HOXA6; HOXA7; HOXA9; HOXA10; HOXA11; HOXA13; EVX1; HIBADH; TAX1BP1; JAZF1; CREB5; CPVL; PRR15; WIPF3; SCRN1; FKBP14; PLEKHA8; NOD1; GGCT; GARS; CRHR2; GHRHR; NEUROD6; CCDC129; C7orf16; PDE1C; LSM5; KBTBD2; RP9P; FKBP9; NT5C3; RP9; BBS9; BMPER; NPSR1; DPY19L1; TBX20; HERPUD2; SEPT7; EEPD1; KIAA0895; ELMO1; TXNDC3; SFRP4; STARD3NL; TRGV7; FAM183B; VPS41; POU6F2; NCRNA00213; GPR141; EPDR1; CHN2; C7orf41; U6; SRP_euk_arch; Y_RNA; 5S_rRNA; hsa-mir-1183; U1; SNORA51; SRP_euk_arch; SRP_euk_arch; snoU13; hsa-mir-550-1; U6; Y_RNA; SNORD93; snoU13; SNORD65; snoU13; SNORA20; snoU13; Y_RNA; U6; hsa-mir-196b; U6; U6; hsa-mir-550-2; 5S_rRNA; SRP_euk_arch; hsa-mir-1200; hsa-mir-548n; hsa-mir-148a; U3
1	0.394736842	200.968753	1.073916	1.444	0.7885	0.05630879	RPS10P7; RPL34P1; CSRP1; TNNI1; PHLDA3; LAD1; PKP1; TNNT2; IGFN1; TMEM9; CACNA1S; KIF21B; C1orf106; GPR25; CAMSAP1L1; KIF14; DDX59; U6
20	0.605263158	58.3471855	3.322891	1.786	0.7643	0.02734413	CTSZ; MTCO2L; C20orf83; MRPS16P; GNASAS; CDH4; C20orf197; CDH26; PPP1R3D; C20orf177; SYCP2; PHACTR3; EDN3; ZNF831; SLMO2; TUBB1; TH1L; GNAS; NPEPL1; STX16; VAPB; RAB22A; PPP4R1L; C20orf85; hsa-mir-296; hsa-mir-646; U7; hsa-mir-298
19	0.447368421	36.908719	0.436184	0.375	0.1482	0.09864681	ZNF382; ZNF529; ZNF566; ZFP82; ZFP14; ZNF146; ZNF565; Y_RNA

GLAD Loss

Chr	Recurrence	Mid Position (Mb)	Reg Size	GDW	Score	pValue	Genes
1	0.342105263	51.275515	0.3891	3	-3.303	0.00153043	CDKN2C; FAF1
9	0.894736842	21.8658185	0.264757	1.5	-3.023	2.46E-044	CDKN2BAS; MTAP; C9orf53; CDKN2A
10	0.710526316	87.624437	0.515356	3	-1.349	0.00583984	GRID1; 5S_rRNA
10	0.763157895	128.053652	0.532908	3	-1.143	0.01068802	C10orf90; 5S_rRNA
10	0.789473684	38.5794035	0.222759	3	-1.052	0.01410177	HSD17B7P2
13	0.526315789	50.575241	3.169932	1.541	-0.464	0.09937283	DLEU7; COX7CP1; DLEU2; FAM10A4; SERPINE3; WDFY2; INTS6; FAM124A; GUCY1B2; RNASEH2B; DLEU1; KCNRG; TRIM13; C13orf1; KPNA3; EBPL; RCBTB1; PHF11; SETDB2; CAB39L; CDADC1; MLNR; FNDC3A; CYSLTR2; RCBTB2; LPAR6; RB1; SRP_euk_arch; hsa-mir-16-1; 5S_rRNA; 5S_rRNA; 5S_rRNA; Y_RNA; hsa-mir-15a; Y_RNA; U6; Y_RNA
14	0.578947368	24.0629455	0.168195	1.5	-0.411	0.09908523	DHRS2; JPH4; THTPA; ZFHX2

Supplementary Table S2. Prioritization by filtering on amplitude (p<0.1), then by modified GTS

HomHMM Gain

Chr	Recurrence	Mid Position (Mb)	Reg Size	GDW	Score	pValue	Genes
7	0.815789474	26.97294675	0.0750125	11	5.5077	0.001	SKAP2
7	0.894736842	54.872287	0.252308	4	4.3118	3.22E-15	SEC61G
20	0.631578947	38.5255555	0.568367	6	2.3968	0.02375686	HSPEP1
20	0.684210526	56.715494	0.103328	5	2.2462	0.00752275	C20orf85
4	0.342105263	54.806086	0.398224	2	1.9598	0.004	GSX2; CHIC2; RPL21P44; snoU13
20	0.684210526	57.0270185	0.036269	4	1.6609	0.01250216	VAPB
3	0.447368421	190.6002995	0.463407	4	1.5059	0.09729489	IL1RAP
7	0.815789474	42.6276285	2.615425	3.077	1.3322	0.003	INHBA; GLI3; PSMA2; C7orf25; MRPL32; HECW1; STK17A; C7orf44; BLVRA; MRPS24; URG4; Y_RNA; U6
8	0.473684211	124.874236	0.506656	3.333	1.275	0.08056952	KLHL38; FAM91A1; FER1L6
20	0.631578947	40.9166945	0.679733	3	1.2711	0.01757728	PTPRT
17	0.421052632	74.47967	0.035492	3	1.2443	0.0888594	RHBDF2
7	0.815789474	21.9276225	2.526969	2.615	1.1833	0.002	SP8; SP4; DNAH11; CDCA7L; RAPGEF5; IL6; TOMM7; FAM126A; KLHL7; hsa-mir-1183; U1; SRP_euk_arch; SNORD93
20	0.605263158	42.5956615	0.065577	3	1.1675	0.03251584	C20orf100
7	0.815789474	91.90800975	0.3552545	2	1.1621	7.49E-05	GATAD1; KRIT1; CYP51A1; hsa-mir-1285-1; Y_RNA
7	0.815789474	46.968329	2.867128	2.4	1.1014	0.001	EPS15L2; SEPT13; IGFBP1; IGFBP3; TNS3; C7orf65; HUS1; PKD1L1; C7orf69; SUNC1; C7orf57; UPP1; U6; U7; U6
20	0.605263158	9.076532	0.765236	3.333	1.0862	0.0627363	PLCB4; PLCB1; SNORA73
5	0.5	9.174911	0.1608	3	1.0799	0.08204215	SEMA5A
7	0.815789474	35.577834	1.032726	2.5	1.0478	0.003	DPY19L2; DPY19L1; TBX20; HERPUD2; SEPT7; U6
8	0.447368421	118.1390945	0.382167	2.667	1.0127	0.09524714	C8orf85; SLC30A8; SRP_euk_arch
20	0.605263158	36.675193	0.094976	2	0.8769	0.01837328	KIAA0406; RPRD1B
7	0.815789474	36.3594515	0.254715	2	0.8696	0.002	EEPD1; KIAA0895; Y_RNA
7	0.815789474	30.1537635	0.653069	1.778	0.838	0.001	ZNRF2; WIPF3; SCRN1; FKBP14; PLEKHA8; NOD1; C7orf41; hsa-mir-550-1; snoU13
7	0.815789474	24.244373	1.812674	1.529	0.6925	0.002	CLK2P; RPS2P32; C7orf30; IGF2BP3; TRA2A; CCDC126; C7orf46; STK31; NPY; MPP6; DFNA5; OSBPL3; snoU13; U6; Y_RNA; snoU13; SNORD65
20	0.631578947	46.516291	0.415958	1.5	0.6681	0.0132602	SRMP1; SULF2; U7; U7
7	0.815789474	44.3963415	0.527537	1.455	0.6501	0.002	POLD2; MYL7; GCK; YKT6; CAMK2B; NUDCD3; NPC1L1; DDX56; TMED4; OGDH; U6
20	0.605263158	60.9074455	0.275059	1.5	0.6412	0.02096412	GATA5; C20orf151; CABLES2; RPS21; LAMA5; OSBPL2; HRH3; GTPBP5
7	0.815789474	33.0358835	0.686869	1.333	0.6238	0.001	KBTBD2; RP9P; FKBP9; NT5C3; RP9; BBS9; 5S_rRNA; SRP_euk_arch; hsa-mir-550-2
17	0.421052632	76.7146585	0.1118125	1.5	0.6126	0.09244294	USP36; CYTH1
17	0.473684211	41.5955355	0.057931	1.5	0.6036	0.0696675	ETV4; DHX8
8	0.473684211	121.5689355	0.070337	1.5	0.5924	0.07361163	MTBP; SNTB1
20	0.605263158	31.6437205	0.030187	1.5	0.5186	0.0515007	BPIL3; C20orf185
20	0.605263158	30.34283725	0.2919315	1	0.4255	0.02146313	COX4I2; BCL2L1; TPX2; MYLK2; FOXS1; DUSP15; TTLL9
7	0.815789474	27.7386355	1.455107	0.833	0.4023	0.001	MIR196B; MIR196B; HOTAIRM1; HOXA11AS; HOXA4; SKAP2; HOXA1; HOXA2; HOXA3; HOXA5; HOXA6; HOXA7; HOXA9; HOXA10; HOXA11; HOXA13; EVX1; HIBADH; TAX1BP1; JAZF1; CREB5; NCRNA00213; hsa-mir-196b; U6
20	0.684210526	44.6253755	0.120483	0.8	0.364	0.00688284	FTLP; PCIF1; ZNF335; MMP9; SLC12A5
20	0.684210526	57.6522155	0.586247	0.909	0.3557	0.01753995	CTSZ; MRPS16P; GNASAS; EDN3; ZNF831; SLMO2; TUBB1; TH1L; GNAS; hsa-mir-296; hsa-mir-298
20	0.631578947	44.155516	0.153866	0.857	0.3265	0.02964721	WFDC6; RPL5P2; SPINLW1; WFDC2; SPINT3; WFDC8; 5S_rRNA
20	0.684210526	58.388277	0.622834	0.857	0.3184	0.02292665	C20orf197; CDH26; PPP1R3D; C20orf177; SYCP2; PHACTR3; U7
20	0.605263158	42.0744925	0.133331	0.667	0.2843	0.02121495	EIF4EBP2P; SFRS6; L3MBTL; U6; Y_RNA; U6
7	0.815789474	38.63389175	1.2872775	0.571	0.2589	0.002	TRGC2; TRGJ2; TRGJP2; TRGJP; TRGJ1; TRGC1; TRGV10; TRGV11; TRGJP1; TRGV9; TRGV5; TRGV8; TRGV4; TRGV3; TRGV2; TRGV1; TRGV5P; TRGVA; TRGV6; TRGVB; SFRP4; STARD3NL; TRGV7; FAM183B; VPS41; POU6F2; EPDR1; SRP_euk_arch
20	0.631578947	37.347778	0.480086	0.625	0.2417	0.02769634	RPS3P2; KIAA1219; C20orf95; SLC32A1; PPP1R16B; FAM83D; SRP_euk_arch; 7SK
20	0.605263158	23.8622755	0.799789	0.6	0.1873	0.07147395	XXyac-YX60D10.1; POM121L3P; CST8; CST9L; CST9; CST3; CST4; CST1; CST2; CST5; GGTLA3; U1; CSTP2; CSTP1; GGTLA3
4	0.342105263	55.3488785	0.197025	0	0	0.00991147	NA
20	0.605263158	29.7962755	0.069531	0	0	0.03298127	NA
20	0.605263158	29.40354	0.102744	0	0	0.06124311	NA

HomHMM Loss

Chr	Recurrence	Mid Position (Mb)	Reg Size	GDW	Score	pValue	Genes
11	0.315789474	109.507784	0.599816	7	-4.166	0.05353168	C11orf87
14	0.631578947	27.5522795	1.365137	10	-2.86	0.06031583	NOVA1
14	0.605263158	23.71335875	0.0316115	7	-1.749	0.09478581	C14orf164
9	0.684210526	22.0808515	1.218563	1.375	-1.7	1.43E-012	CDKN2BAS; MTAP; IFNE; C9orf53; CDKN2A; CDKN2B; DMRTA1; hsa-mir-31
10	0.815789474	98.9062815	0.219007	5	-1.696	0.00973066	SLIT1
13	0.605263158	50.975263	0.314958	6	-1.641	0.07650798	DLEU1
11	0.315789474	104.2106395	1.309271	2.6	-1.464	0.06308541	CASP5; CASP4; CASP12; DDI1; PDGFD
11	0.368421053	107.814429	0.105122	2.5	-1.406	0.03849318	RAB39; SLC35F2
14	0.684210526	38.7609815	0.088249	5	-1.298	0.06337425	CLEC14A
13	0.605263158	26.106365	0.10441	5	-1.252	0.09425172	U6
11	0.368421053	113.113998	0.171082	2.5	-1.109	0.07901189	TTC12; NCAM1
10	0.815789474	130.0023575	0.407323	2.5	-0.974	0.00376887	MKI67; PTPRE
11	0.368421053	116.622076	0.131638	2	-0.805	0.09891034	ZNF259; BUD13
13	0.631578947	26.4066385	0.424403	2.5	-0.689	0.06712056	TMEM46; Y_RNA
13	0.605263158	26.81138375	0.1727815	2	-0.51	0.09044853	RNF6; CDK8
14	0.657894737	24.095536	0.100012	1.5	-0.47	0.03921956	DHRS2; JPH4
14	0.605263158	23.8316125	0.044125	1	-0.27	0.07939416	MYH6; CMTM5; IL25; EFS; SLC22A17
10	0.815789474	88.609891	0.393474	0.6	-0.242	0.00282496	FAM25A; C10orf116; SNCG; MMRN2; BMPR1A; LDB3; OPN4; BMS1P3; U6; U1
14	0.605263158	20.92809625	0.1734885	0.857	-0.223	0.0868453	RNASE10; NP; TMEM55B; OSGEP; KLHL33; TEP1; 5S_rRNA
14	0.605263158	26.06211	0.153492	0	0	0.0744605	NA
9	0.5	31.619394	0.524102	0	0	0.07848185	NA

Supplementary Table S2. Prioritization by filtering on amplitude (p<0.1), then by modified GTS

BioHMM Gain

Chr	Recurrence	Mid Position (Mb)	Reg Size	GDW	Score	pValue	Genes
7	0.894736842	54.897592	0.302918	5	6.1357	0.0000	SEC61G
7	0.868421053	26.9828035	0.056557	10	4.4991	0.0012	SKAP2
5	0.5	38.163874	0.614326	8	3.1855	0.0654	EGFLAM
20	0.710526316	57.9437595	0.135107	7	2.635	0.0201	EDN3
4	0.342105263	54.806086	0.398224	2	1.7573	0.0104	GSX2; CHIC2; RPL21P44; snoU13
7	0.842105263	29.851105	0.047752	3	1.3493	0.0017	WIPF3
7	0.842105263	48.1911105	0.148341	3	1.3378	0.0018	UPP1
7	0.842105263	32.3211055	0.101359	3	1.3316	0.0019	PDE1C
7	0.842105263	91.93295975	0.3053545	1.6	0.8729	0.0002	GATAD1; KRIT1; CYP51A1; hsa-mir-1285-1; Y_RNA
7	0.842105263	30.97274125	0.3607235	1.667	0.7581	0.0015	FAM188B; INMT; GHRHR
7	0.842105263	30.6531605	0.168505	1.5	0.6894	0.0014	GARS; CRHR2
1	0.368421053	200.937621	0.147022	1.25	0.6846	0.0628	CACNA1S; KIF21B; C1orf106; U6
7	0.842105263	30.1675495	0.391869	1.5	0.6773	0.0016	ZNRF2; SCRN1; FKBP14; PLEKHA8; C7orf41; hsa-mir-550-1
3	0.421052632	193.63141	4.740988	1.447	0.5957	0.0956	SDHALP1; VEZF1L1; TCTEX1D2; PCYT1A; Osta; ZDHHC19; TFRC; TNK2; MUC4; SDHALP2; PPP1R2; FAM43A; C3orf21; LSG1; TMEM44; GP5; HES1; CPN2; LRRC15; OPA1; HRASLS; C3orf59; FGF12; MUC20; Y_RNA; Y_RNA; U2; SRP_euk_arch; Y_RNA; SRP_euk_arch; U6; SRP_euk_arch; U1; snoU13; Y_RNA; hsa-mir-570; Y_RNA; 7SK
7	0.842105263	33.0358835	0.686869	1.333	0.5954	0.0018	KBTBD2; RP9P; FKBP9; NT5C3; RP9; BBS9; 5S_rRNA; SRP_euk_arch; hsa-mir-550-2
3	0.447368421	190.3889783	1.5647535	1.091	0.4303	0.0914	CCDC50; UTS2D; OSTN; IL1RAP; TMEM207; CLDN16; CLDN1; LEPREL1; TP63; 7SK; U6
1	0.368421053	201.4081365	0.195149	0.667	0.3561	0.0678	RPS10P7; CSRP1; TNNI1; PHLDA3; LAD1; TNNT2
7	0.842105263	47.139409	0.17921	0	0	0.0016	NA
4	0.342105263	55.3488785	0.197025	0	0	0.0182	NA
1	0.315789474	199.627756	0.036042	0	0	0.0993	NA

BioHMM Loss

Chr	Recurrence	Mid Position (Mb)	Reg Size	GDW	Score	pValue	Genes
10	0.842105263	9.441817	2.371964	5.5	-2.185	0.0034	Y_RNA; 5S_rRNA
9	0.894736842	21.8658185	0.264757	1.5	-2.129	2.90E-024	CDKN2BAS; MTAP; C9orf53; CDKN2A
10	0.815789474	12.008937	0.146134	4	-1.662	0.0031	UPF2
11	0.421052632	113.2765165	0.153955	3	-1.421	0.0508	DRD2; TTC12
10	0.842105263	26.425852	0.147118	3	-1.313	0.0015	MYO3A
10	0.842105263	11.5507215	0.278669	3	-1.202	0.0032	USP6NL
10	0.842105263	2.1836455	0.447867	3	-1.165	0.0041	U6
10	0.815789474	12.3471535	0.190893	2.5	-1.084	0.0021	CAMK1D; CDC123
10	0.815789474	19.6262475	0.813461	2.5	-1.02	0.0036	C10orf112; U3
10	0.868421053	87.7201785	0.323873	2.5	-0.936	0.0043	GRID1; 5S_rRNA
10	0.815789474	26.9705255	0.321065	2	-0.887	0.0017	C10orf50; PDSS1; SNORA57; U6
4	0.473684211	92.493808	0.752692	2	-0.762	0.0685	FAM190A; 5S_rRNA
10	0.815789474	29.10892	0.201274	1.667	-0.719	0.0022	C10orf126; U6; 5S_rRNA
10	0.815789474	12.7528015	0.197845	1.5	-0.673	0.0016	CAMK1D; MIR548Q
10	0.815789474	24.15062425	1.6267945	1.273	-0.563	0.0018	PRINS; MSRB2; PTF1A; C10orf115; C10orf67; OTUD1; KIAA1217; snoU13; 5S_rRNA; U4; hsa-mir-603
10	0.842105263	25.399507	0.438356	1.25	-0.53	0.0020	PRTFDC1; ENKUR; THNSL1; GPR158
10	0.815789474	16.507721	0.333412	1	-0.415	0.0031	PTER; C1QL1; RSU1; snoU13; U2
10	0.842105263	1.20367175	0.2389245	1	-0.39	0.0039	C10orf139; C10orf110; WDR37; IDI1; U7
14	0.605263158	24.06394125	0.1662035	1.25	-0.385	0.0624	DHRS2; JPH4; THTPA; ZFHX2
10	0.815789474	0.7770605	0.519637	0.75	-0.306	0.0036	GTPBP4; LARP4B; C10orf108; DIP2C
10	0.815789474	28.253949	0.124994	0	0	0.0018	NA
10	0.868421053	133.6221105	0.044281	0	0	0.0037	NA
14	0.605263158	38.7956555	0.045137	0	0	0.0883	NA
13	0.736842105	18.7022315	0.200997	0	0	0.0914	NA

Supplementary Table S3. Prioritization by modified GTS only

BioHMM GAINS

Chr	Reccurence	Mid Pos. Mb	Reg. Sz Mb	GDW	Score	pValue	Genes
7	0.894736842	54.897592	0.302918	5	6.135707298	0	SEC61G
7	0.868421053	26.9828035	0.056557	10	4.499074861	0.001244756	SKAP2
5	0.5	38.163874	0.614326	8	3.185516747	0.065407564	EGFLAM
17	0.421052632	44.0331385	0.049955	9	3.078864569	0.140968987	MAPT
16	0.210526316	54.73646475	0.4884815	11	2.982203156	0.348649113	IRX5
1	0.315789474	60.098707	0.303306	8	2.856783418	0.203572691	FGGY
7	0.842105263	41.5863995	0.532967	6	2.661678344	0.001931064	INHBA
20	0.710526316	57.9437595	0.135107	7	2.63500168	0.020134047	EDN3
16	0.236842105	64.497578	0.973198	11	2.365018914	0.366795312	CDH11
7	0.815789474	14.1569875	1.481851	5.5	2.229129814	0.005385366	ETV1; DGKB

BioHMM LOSS

Chr	Reccurence	Mid Pos. Mb	Reg. Sz Mb	GDW	Score	pValue	Genes
10	0.842105263	9.441817	2.371964	5.5	-2.184596896	0.003422657	Y_RNA; 5S_rRNA
9	0.894736842	21.8658185	0.264757	1.5	-2.129226053	2.90E-024	CDKN2BAS; MTAP; C9orf53; CDKN2A
10	0.815789474	123.8785645	0.191387	5	-1.809164504	0.008337856	TACC2
10	0.815789474	131.2748623	0.2375915	5	-1.793255326	0.008815562	MGMT
10	0.815789474	131.8828615	0.179833	5	-1.771502164	0.009507491	GLRX3
1	0.315789474	5.586057	0.845456	8	-1.666942593	0.280374987	NPHP4
10	0.815789474	12.008937	0.146134	4	-1.662095424	0.003073859	UPF2
10	0.815789474	113.2272825	0.929945	5	-1.652502406	0.014194943	SHOC2
15	0.342105263	38.543396	0.143662	5	-1.511546818	0.189719901	SPRED1
11	0.421052632	113.2765165	0.153955	3	-1.420764292	0.050796006	DRD2; TTC12

DNAcopy GAINS

Chr	Reccurence	Mid Pos. Mb	Reg. Sz Mb	GDW	Score	pValue	Genes
5	0.447368421	38.163874	0.614326	8	3.611011765	0.062138613	EGFLAM
17	0.421052632	44.0216635	0.072905	10	3.3273125	0.147488247	MAPT
16	0.210526316	64.497578	0.973198	11	2.119825	0.397855816	CDH11
4	0.315789474	54.806086	0.398224	2	2.10759375	0.005033477	GSX2; CHIC2; RPL21P44; snoU13
16	0.210526316	52.918444	0.427168	5	1.7592875	0.300036635	CHD9
2	0.210526316	85.7085575	0.054621	5	1.449075	0.336938781	Y_RNA
8	0.421052632	124.86118	0.532768	3.666666667	1.342	0.12338597	KLHL38; FAM91A1; FER1L6
21	0.289473684	41.827567	0.713304	6	1.305	0.331326399	DSCAM; SNORA51
16	0.210526316	85.9879645	0.072619	7	1.2886125	0.403414163	IRF8
7	0.815789474	15.6302965	2.731487	3	1.151418433	0.007809723	DGKB; TMEM195; MEOX2; ISPD; SOSTDC1; BZW2; TSPAN13

DNAcopy LOSS

Chr	Reccurence	Mid Pos. Mb	Reg. Sz Mb	GDW	Score	pValue	Genes
11	0.315789474	109.521102	0.626452	8	-4.163816667	0.086221096	C11orf87
14	0.5	34.26139775	1.1114165	11.5	-2.874110526	0.146329629	EGLN3; NPAS3
1	0.342105263	51.275515	0.3891	3	-2.819550306	0.004535859	CDKN2C; FAF1
9	0.894736842	21.8658185	0.264757	1.5	-2.644066176	1.86E-036	CDKN2BAS; MTAP; C9orf53; CDKN2A
13	0.5	89.109463	1.937604	10	-2.229215789	0.172236056	SLITRK5
11	0.315789474	107.5300975	0.820415	4	-2.177119444	0.077232038	CUL5; RAB39; SLC35F2; SLN; ELMOD1; CWF19L2
15	0.342105263	38.6268415	0.149003	6	-2.068484615	0.159457972	SPRED1
18	0.315789474	53.680115	0.780252	6	-1.614	0.231063548	TCF4; SNORA73
13	0.578947368	49.357849	0.369072	4.5	-1.334384091	0.077464724	CYSLTR2; 5S_rRNA
10	0.710526316	49.654245	0.748988	3.75	-1.309372222	0.021298479	FRMPD2; MAPK8; WDFY4; PTPN20C

GLAD GAINS

Chr	Reccurence	Mid Pos. Mb	Reg. Sz Mb	GDW	Score	pValue	Genes
7	0.894736842	54.897592	0.302918	5	6.544693745	0	SEC61G
16	0.210526316	52.601305	1.061446	8	2.292722543	0.346421951	CHD9; TOX3
4	0.315789474	54.806086	0.398224	2	2.055472664	0.007669755	GSX2; CHIC2; RPL21P44; snoU13
16	0.210526316	46.0134325	1.681609	6	1.955080385	0.323306218	SHCBP1; VPS35; ORC6L; MYLK3; C16orf87; U6
5	0.447368421	32.165986	0.03893	3	1.549262499	0.044003304	GOLPH3
2	0.210526316	85.703029	0.043564	4	1.354749939	0.315885429	Y_RNA
16	0.210526316	85.9879645	0.072619	7	1.291722278	0.4090546	IRF8
5	0.421052632	44.7109645	1.940599	2.8	1.220523414	0.089920817	HCN1; MRPS30; FGF10; SRP_euk_arch; U6
12	0.210526316	56.3739965	0.236025	1.6	1.155743928	0.135759071	WIBG; DGKA; SILV; CDK2; RAB5B; SUOX; IKZF4; RPS26; ERBB3; U1
5	0.447368421	37.89945675	1.1431605	2.5	1.109090299	0.072707077	EGFLAM; GDNF; WDR70; NUP155; U6; U6

GLAD LOSS

Chr	Reccurence	Mid Pos. Mb	Reg. Sz Mb	GDW	Score	pValue	Genes
15	0.315789474	35.898995	1.438628	17	-4.764332207	0.227192961	ZNF770
1	0.342105263	51.275515	0.3891	3	-3.303320705	0.001530432	CDKN2C; FAF1
9	0.894736842	21.8658185	0.264757	1.5	-3.02311909	2.46E-044	CDKN2BAS; MTAP; C9orf53; CDKN2A
13	0.5	89.0293995	1.777477	9	-2.004923863	0.177775815	SLITRK5
12	0.315789474	112.1404285	0.104943	6	-1.41780221	0.260593596	BRAP
10	0.710526316	87.624437	0.515356	3	-1.348667283	0.005839841	GRID1; 5S_rRNA
18	0.315789474	53.5749525	0.569927	4.5	-1.316987592	0.218219695	TCF4; SNORA73
18	0.315789474	62.190902	7.588336	2.838709677	-1.163207811	0.144063644	SERPINB10; SERPINB2; C18orf20; DSEL; CDH19; CDH7; SERPINB8; HMSD; SERPINB7; SERPINB3; SERPINB11; SERPINB4; SERPINB13; SERPINB12; SERPINB5; VPS4B; KDSR; BCL2; PHLPP1; ZCCHC2; TNFRSF11A; KIAA1468; PIGN; RNF152; CDH20; U6; Y_RNA; snoU13; U6; SRP_euk_arch; U6
10	0.763157895	128.053652	0.532908	3	-1.142848307	0.010688024	C10orf90; 5S_rRNA
16	0.289473684	6.3711785	1.278965	7	-1.130984353	0.334468006	U6

HomHMM GAINS

Chr	Reccurence	Mid Pos. Mb	Reg. Sz Mb	GDW	Score	pValue	Genes
7	0.815789474	26.97294675	0.0750125	11	5.50773086	0.000564208	SKAP2
7	0.894736842	54.872287	0.252308	4	4.3118042	3.22E-015	SEC61G
20	0.631578947	38.5255555	0.568367	6	2.396836781	0.02375686	HSPEP1
20	0.684210526	56.715494	0.103328	5	2.246241517	0.007522745	C20orf85
4	0.342105263	54.806086	0.398224	2	1.959755399	0.003924166	GSX2; CHIC2; RPL21P44; snoU13
8	0.447368421	118.9420263	0.4998705	5	1.794454068	0.108811851	EXT1
3	0.421052632	188.9878415	0.422959	6	1.727071059	0.180984664	TPRG1
20	0.684210526	57.0270185	0.036269	4	1.660945208	0.012502158	VAPB
1	0.342105263	157.4342903	0.1960175	7	1.624275976	0.284234799	FCRL5
1	0.315789474	148.579321	0.043796	4	1.517512071	0.183996884	NBPF15

HomHMM Loss

Chr	Reccurence	Mid Pos. Mb	Reg. Sz Mb	GDW	Score	pValue	Genes
11	0.315789474	109.507784	0.599816	7	-4.165729502	0.053531675	C11orf87
14	0.631578947	27.5522795	1.365137	10	-2.860282792	0.060315826	NOVA1
14	0.605263158	23.71335875	0.0316115	7	-1.748792132	0.094785808	C14orf164
9	0.684210526	22.0808515	1.218563	1.375	-1.700426409	1.43E-012	CDKN2BAS; MTAP; IFNE; C9orf53; CDKN2A; CDKN2B; DMRTA1; hsa-mir-31
10	0.815789474	98.9062815	0.219007	5	-1.696248759	0.009730665	SLIT1
12	0.421052632	78.5139695	0.141163	6	-1.66799518	0.150794111	NAV3
13	0.605263158	50.975263	0.314958	6	-1.641197221	0.076507979	DLEU1

11	0.342105263	117.603889	0.058658	5	-1.583738343	0.168001304	DSCAML1
11	0.315789474	104.2106395	1.309271	2.6	-1.464398825	0.063085413	CASP5; CASP4; CASP12; DDI1; PDGFD
11	0.368421053	107.814429	0.105122	2.5	-1.405672999	0.038493176	RAB39; SLC35F2

Supplementary Table S4. The final set of 35 MRIs identified from the consolidated data

Rated By	Method	CNA	Chr	Start	End	Gene
A	B, G	Gain	1	201.31	201.51	RPS10P7; CSRP1; TNNI1; PHLDA3; LAD1; TNNT2
A	D, G	Gain	3	183.13	185.38	HSP90AA5P; IGF2BP2; SENP2; LIPH; TMEM41A; MAP3K13; C3orf70; EHHADH; MAGEF1; VPS8; EPHB3; CHRD; THPO; POLR2H; CLCN2; FAM131A; EIF4G1; CAMK2N2; PSMD2; ECE2; VWA5B2; DVL3; EIF2B5; HTR3E; HTR3C; HTR3D; PARL; MAP6D1; YEATS2; KLHL6; KLHL24; MCF2L2; Y_RNA; SNORA63; snoU13; SNORA63; hsa-mir-1224; SNORD66
A	B, D	Gain	3	189.61	190.99	UTS2D; OSTN; IL1RAP; TMEM207; CLDN16; CLDN1; LEPREL1; TP63; 7SK; U6
A,B	B, D, G, H	Gain	4	54.61	55.01	**GSX2; CHIC2;** RPL21P44; snoU13
A,B	G, D, B	Gain	5	37.86	38.47	EGFLAM
A	D, G	Gain	5	43.74	44.85	MRPS30; FGF10; SRP_euk_arch; U6
A,B	H, D, B, G	Gain	7	20.89	21.63	SP4; DNAH11; hsa-mir-1183; U1; SRP_euk_arch
A,B	H, D, B, G	Gain	7	23.6	24.1	CLK2P; CCDC126; C7orf46; STK31; snoU13
A,B	H, B	Gain	7	26.94	27.01	SKAP2
A,B	H, B	Gain	7	29.97	30.36	ZNRF2; SCRN1; FKBP14; PLEKHA8; C7orf41; hsa-mir-550-1
A	H, B	Gain	7	32.69	33.38	KBTBD2; RP9P; FKBP9; NT5C3; RP9; BBS9; 5S_rRNA; SRP_euk_arch; hsa-mir-550-2
A	H, B	Gain	7	35.06	36.09	DPY19L2; DPY19L1; TBX20; HERPUD2; SEPT7; U6
A	H, B	Gain	7	37.99	39.28	TRGC2; TRGJ2; TRGJP2; TRGJP; TRGJ1; TRGC1; TRGV10; TRGV11; TRGJP1; TRGV9; TRGV5; TRGV8; TRGV4; TRGV3; TRGV2; TRGV1; TRGV5P; TRGVA; TRGV6; TRGVB; SFRP4; STARD3NL; TRGV7; FAM183B; VPS41; POU6F2; EPDR1; SRP_euk_arch
A	H,B	Gain	7	41.32	41.85	INHBA
A	B,D,G,H	Gain	7	54.75	55.05	**SEC61G**, **NA***
A,B	B,D,G,H	Gain	7	91.73	92.09	GATAD1; KRIT1; CYP51A1; hsa-mir-1285-1; Y_RNA
B	G,D	Gain	16	52.7	53.13	CHD9
B	G,D	Gain	16	85.95	86.02	IRF8
A	D, H	Gain	20	23.46	24.26	POM121L3P; CST8; CST9L; CST9; CST3; CST4; CST1; CST2; CST5; GGTLA3; U1; CSTP2; CSTP1; GGTLA3
A,B	D,G,H,B	Gain	20	57.88	58.01	EDN3
A,B	D, G	Loss	1	51.08	51.47	**CDKN2C; FAF1**
A,B	B,D,G,H	Loss	9	21.73	22	**CDKN2BAS**; MTAP; C9orf53; **CDKN2A**
A,B	B, D	Loss	10	11.41	11.69	USP6NL
A,B	D, G, B	Loss	10	87.37	87.88	**GRID1**; 5S_rRNA
A	B, D	Loss	10	122.32	124.03	RPS15AP5; TACC2; NSMCE4A; FGFR2; C10orf85; BRWD2; PPAPDC1A; BTBD16; Y_RNA; 7SK
A	B, D, G, H	Loss	10	127.79	130.21	C10orf90; 5S_rRNA; MKI67; PTPRE
A	H, D	Loss	11	103.73	104.87	CASP5; CASP4; CASP12; DDI1; PDGFD
A,B	H, D	Loss	11	107.76	107.87	RAB39; SLC35F2
A,B	H, D	Loss	11	109.21	109.83	C11orf87
A	H, B, D	Loss	11	113.03	116.69	DRD2; TTC12; ZNF259; BUD13; NCAM1
A	H, B	Loss	13	26.54	26.72	RNF6; TMEM46; CDK8
A	G, D, H	Loss	13	50.82	51.13	DLEU1
A	B, G, H	Loss	14	24.05	24.15	DHRS2; JPH4
A	D, H	Loss	14	26.87	28.23	NOVA1
B	B,D	Loss	15	38.47	38.62	SPRED1

Supplementary Table S5: Regions of interest identified by GISTIC

	**BioHMM**			**DNAcopy**		
	**Wide peaks Mb**	**% CNA**	**# Genes**	**Wide peaks Mb**	**% CNA**	**# Genes**
**Gain**	106.8	3.34	359	44.1	1.38	154
**Loss**	16.5	0.52	115	64.7	2.02	272
**Total**	123.3	3.86	474	108.8	3.4	426
**Gain:Loss**		**6.4**			**0.7**	
	**GLAD**			**HomHMM**		
	**Wide peaks Mb**	**% CNA**	**# Genes**	**Wide peaks Mb**	**% CNA**	**# Genes**
**Gain**	26.8	0.84	249	7.8	0.24	85
**Loss**	87.6	2.74	414	4.1	0.13	46
**Total**	114.4	3.58	663	11.9	0.37	131
**Gain:Loss**		**0.3**			**1.8**	
